# Physiology of Ovarian Development in Crustaceans: Interactions Among Hormones, Nutrients, and Environmental Factors From Integrated Perspectives

**DOI:** 10.1155/anu/4900891

**Published:** 2025-12-01

**Authors:** Tariq Dildar, Wenxiao Cui, Hongyu Ma

**Affiliations:** ^1^Guangdong Provincial Key Laboratory of Marine Biotechnology, College of Science, Shantou University, Shantou 515063, China; ^2^International Joint Research Center for the Development and Utilization of Important Mariculture Varieties Surrounding the South China Sea Region, College of Science, Shantou University, Shantou 515063, China; ^3^STU-UMT Joint Shellfish Research Laboratory, College of Science, Shantou University, Shantou 515063, China

**Keywords:** crustaceans, environmental factors, hormones, nutrition, ovarian development

## Abstract

Reproductive maturation remains a central bottleneck in crustacean aquaculture, as ovarian development dictates broodstock quality, fecundity, and larval viability. This review synthesizes current knowledge on the physiological regulation of ovarian maturation in decapod crustaceans, emphasizing the interplay of hormones, nutrients, and environmental factors. Eyestalk-derived neuropeptides of the crustacean hyperglycemic hormone (CHH) family, together with molt-inhibiting hormone (MIH), vitellogenesis-inhibiting hormone (VIH), methyl farnesoate (MF), and ecdysteroids, constitute the core hormonal regulators, with CHH and MF promoting vitellogenesis while VIH exerts inhibitory control. Among nutritional drivers, proteins, amino acids (notably arginine), long-chain polyunsaturated fatty acids (LC-PUFAs), cholesterol, and carotenoids exert the most pronounced effects on vitellogenesis, oocyte maturation, and larval quality, with their regulatory roles frequently mediated through endocrine pathways such as mTOR and steroidogenesis. Evidence across species indicates that optimal ovarian growth is generally achieved at dietary protein levels of 30%–35%, lipid levels of 8%–12%, and specific amino acid concentrations including 2.5%–4.5% arginine, 0.6%–1.0% taurine, and 1.5%–1.7% threonine. Favorable temperature and photoperiod can regulate ovarian development better and lead to higher spawning rates and reproductive effects compared to salinity. Finally, the economic feasibility of nutritional interventions is evaluated, highlighting that while cholesterol and krill oil are biologically effective, their high cost necessitates strategic use alongside sustainable alternatives such as phytosterols, marine by-products, and plant- or insect-based proteins. By consolidating hormonal, nutritional, and environmental perspectives, this review outlines regulatory mechanisms of ovarian development while identifying practical strategies to improve broodstock management and reproductive efficiency in crustacean aquaculture.

## 1. Introduction

Crustaceans constitute a critical component of global food security, providing high-quality protein and essential micronutrients to human populations [1]. Global aquaculture production has expanded rapidly in recent decades, with crustacean cultivation emerging as a cornerstone sector, particularly for high-value decapod species [2]. As of 2022, global aquaculture production of decapod crustaceans had expanded to 12.7 million tonnes, worth approximately USD 75.7 billion, with Asia leading worldwide production [3]. Among cultured species, the whiteleg shrimp (*Penaeus vannamei*) accounts for the largest production, yielding ~5.7 million tonnes, or 44% of the decapod total production. The red swamp crayfish (*Procambarus clarkii*) ranks second, with 2.6 million tonnes representing about 20% of the total. Other commercially significant species include the giant tiger prawn (*Penaeus monodon*), Chinese mitten crab (*Eriocheir sinensis*), giant river prawn (*Macrobrachium rosenbergii*), American lobster (*Homarus americanus*), ornate spiny lobster (*Panulirus ornatus*), and various mud crabs (*Scylla spp*.) [4]. Decapod crustaceans also represent a major component of global capture fisheries, with total landings reaching 5.3 million tonnes in 2022, underscoring their continued economic and nutritional importance alongside aquaculture production [4]. Marine waters accounted for the majority of crustacean fisheries, 94.0%, while aquaculture contributed 67.9% of all crustacean production. Although inland fisheries were not a major source of crustaceans, inland aquaculture accounted for ~40% of total farmed crustaceans.

In decapod crustaceans, reproductive maturation is a fundamental biological process with direct implications for aquaculture, and in females, it is primarily characterized by the sequential stages of ovarian development [5]. Ovarian development, a pronounced determinant of reproductive success, is regulated through a sophisticated interplay of hormones, nutrients, and environmental factors [6, 7]. Among the neuroendocrine factors regulating crustacean reproduction, the eyestalk-derived neuropeptides of the crustacean hyperglycemic hormone (CHH) family, which includes key hormones such as CHH, molt-inhibiting hormone (MIH), vitellogenesis-inhibiting hormone (VIH), ecdysteroids, and methyl farnesoate (MF), mediate physiological trade-offs among growth, molting, and reproductive processes [8, 9]. Within this family, VIH has been of particular interest due to its inhibitory effects on vitellogenesis, acting either by suppressing vitellogenesis in target tissues or by limiting protein uptake on developing oocytes [9]. In addition to eyestalk-derived peptides, the neuroendocrine regulation of crustacean reproduction is modulated by several auxiliary factors, including biogenic amine neurotransmitters, as well as ecdysteroids and the sesquiterpenoid MF, synthesized and secreted by the Y-organs and mandibular organs, respectively [10, 11]. Reproductive development in crustaceans is highly dependent on adequate nutritional intake, as the diet of broodstock directly influences the biochemical composition of the ovary, hepatopancreas, and muscle in crustaceans. These nutritional effects not only determine the species' commercial quality but also play a critical role in regulating ovarian maturation [11]. Among these nutrients, amino acids influence flavor quality and play a direct role in the synthesis of reproductive proteins as well as in immune responses [12]. Fatty acids, long-chain polyunsaturated fatty acids (LC-PUFAs) and monounsaturated fatty acids (MUFAs), enhance crab quality and are key determinants of gonadal maturation, fecundity, and larval hatching success in crustaceans [13]. In addition, carotenoids influence tissue pigmentation, a critical indicator of commercial value in crustaceans [14]. They also accumulate in oocytes as essential components of major yolk proteins, serving as important energy substrates during ovarian maturation and early embryonic development [15]. The molecular mechanisms underlying nutrient mobilization during reproduction, such as lipoprotein-mediated transport of carotenoids and LC-PUFAs through lipid transfer proteins, have not been fully elucidated, thereby constraining the development of optimized dietary formulations in aquaculture practices [16]. Exposure to environmental factors, particularly thermal regimes, salinity fluctuations, and photoperiodic cycles, exerts dose-dependent effects on vitellogenesis by altering neuroendocrine outputs and metabolic resource allocation [16]. However, the synergistic interactions between abiotic stressors under environmental change and their cumulative impacts on ovarian development are still poorly understood.

The objective of this review is to evaluate current knowledge on the regulation of ovarian development in crustaceans, with emphasis on the roles of hormones, nutrition, and environmental conditions. Special attention is given to the influence of key hormones such as CHH, MIH, VIH, ecdysteroids, and MF, as well as to the contributions of nutrients including proteins, amino acids, lipids, fatty acids, and carotenoids in supporting vitellogenesis and oocyte maturation. The effects of environmental factors such as temperature, photoperiod, salinity, and ocean warming are discussed in relation to their impact on reproductive physiology, along with the nutritional intervention and economic implications of dietary supplementation. By integrating these perspectives, this paper aims to provide a consolidated framework to support improved broodstock management and future strategies for sustainable crustacean aquaculture.

## 2. Complexity and Dynamics of Ovarian Development in Crustaceans

Ovarian development in crustaceans follows a progressive sequence of morphological and histological changes, though the specific staging patterns vary considerably among species [17, 18]. The process begins with germline cell proliferation, where oogonia undergo mitotic division to form primary oocytes. This is followed by previtellogenesis, characterized by oocyte growth and increased ribosomal activity, and vitellogenesis, where yolk proteins are synthesized and deposited. The final stage of oocyte maturation prepares the gametes for fertilization. These developmental processes are regulated by a complex interplay of endocrine factors, including eyestalk neuropeptides (CHH, VIH, MIH), MF, 20-hydroxyecdysone, and various biogenic amines [19]. While the fundamental biological processes are conserved across species, the classification of ovarian stages differs significantly. Many crab species, including the mud crab (*Scylla paramamosain*), swimming crab (*Portunus pelagicus*), and Chinese mitten crab (*E. sinensis*), exhibit five distinct ovarian stages. These typically progress from predevelopmental stages to mature stages, with visible changes in ovarian color and oocyte morphology [20–22]. However, other crabs demonstrate more complex six-stage patterns, such as freshwater crab (*Sinopotamon henanense*), blue crab (*Calinectes danae*), and brown shrimp (*Penaeus subtilis*) [23, 24] or simpler four-stage systems, including Iranian freshwater crab (*Sodhiana iranica*), and giant mud crab (*Scylla olivacea*) [25, 26]. These variations reflect species-specific reproductive strategies and adaptations to life histories.

In penaeid shrimps, ovarian maturation is prominently marked by a series of vivid color changes, providing a reliable external indicator of developmental progress. In *P. monodon* from Malaysia, four ovarian stages were morphologically identified by color (yellow, green–yellowish, light green, and dark green) and confirmed histologically by the presence of perinucleolar, yolkless, and yolky oocytes, with the gonadosomatic index (GSI) increasing significantly with each advancing stage [27]. Beyond penaeids, prawn groups exhibit similar yet distinct patterns. The ridgetail white prawn (*Exopalaemon carinicauda*) displays five ovarian stages under laboratory conditions, with the ovary changing from white to yellow [28]. Furthermore, the *P. clarkii* follows a four-stage histological development sequence from previtellogenic to mature [29].

Despite these variations among staging systems, common morphological features are evident across most crustaceans. Early developmental stages are typically marked by translucent, ribbon-like ovarian tissue containing oogonia and previtellogenic oocytes. The ovary often adopts orange or yellow pigmentation as vitellogenesis progresses, eventually becoming darkly colored (purple or deep orange) when filled with mature oocytes. Microscopic examination reveals consistent cellular changes throughout development, including oocyte enlargement, chromatin pattern modifications, and lipid vesicle accumulation [13]. Some species incorporate a distinct recovery phase after spawning, while others immediately begin a new reproductive cycle.

The progression of ovarian development is influenced by numerous internal and external factors, including hormonal regulation, environmental conditions (e.g., temperature, photoperiod), and nutrient availability [21]. In an aquaculture environment, understanding these developmental patterns and their controlling factors is crucial for optimizing reproductive management. Aquaculture strategies can significantly improve reproductive efficiency and larval production outcomes by aligning environmental conditions and nutritional inputs with species-specific ovarian development stages. These perspectives are particularly valuable for commercially important crustacean species, where controlled reproduction remains a challenge.

## 3. Regulatory Mechanisms of Hormones Modulate Ovarian Development in Crustaceans

**Figure 1:**
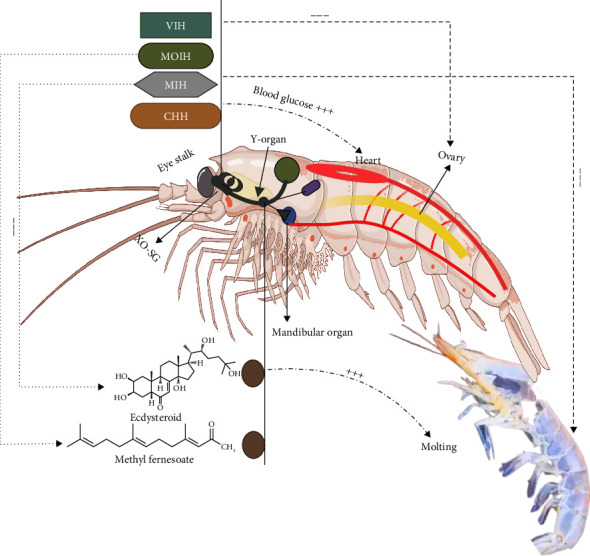
Neuroendocrine regulation of molting and reproduction in crustaceans. This diagram highlights the roles of key hormones vitellogenesis-inhibiting hormone (VIH), molt-inhibiting hormone (MIH), mandibular organ-inhibiting hormone (MOIH), and crustacean hyperglycemic hormone (CHH) in regulating vital processes such as vitellogenesis, molting, and ovarian development. The illustration shows the interaction between the eyestalk complex, central nervous system, and Y-organ, demonstrating how environmental factors like light and temperature influence hormone secretion. These hormonal signals coordinate reproductive processes, integrating external environmental cues with the molting cycle and ovarian maturation.

Within the central nervous system of crustaceans, particularly in the cerebral and thoracic ganglia, certain neurons act as neuroendocrine cells, releasing both neurotransmitters and neurohormones. In decapods that possess eyestalks, this structure represents the primary site of hormone release. Comparative studies have since shown that the two principal neurosecretory systems of decapods, the eyestalk-associated X-organ sinus gland (XO-SG) complex and the pericardial organ, are also present across other crustacean groups [30]. However, studies mainly focused on the XO-SG [31, 32]. The XO is composed of neurosecretory cells that synthesize various hormones, which are stored and secreted by the SG, a neurohemal organ situated near a blood sinus. The SG facilitates the controlled release of hormones in response to environmental and internal stimuli, thereby regulating physiological processes like energy regulation and molting [33, 34]. Hormones secreted from the XO-SG system perform a central role in regulating diverse physiological functions, including metabolic adaptation, molting, modulation of immune activity, and gonadal maturation in response to environmental fluctuations [30]. As illustrated in Figure 1, the XO-SG system acts as a central command hub, releasing members of the CHH family containing hormones such as CHH, MIH, mandibular organ-inhibiting hormone (MOIH), and VIH that exert downstream effects on the Y-organs and mandibular organs.

The hormones produced by the XO–SG complex primarily belong to the CHH family, which includes CHH, MIH, MOIH, and VIH [35]. CHH is responsible for maintaining blood glucose levels and metabolic balance, while MIH controls ecdysteroid synthesis in the Y-organs, a process critical for initiating molting [36]. In female crustaceans, VIH functions as a key regulator of reproductive physiology by inhibiting vitellogenesis and thus controlling oocyte maturation. MOIH impacts larval development and reproductive behavior by inhibiting MF synthesis in the mandibular organs [37]. The Y-organs, situated in the cephalothorax, produce ecdysteroids, such as ecdysone and 20-hydroxyecdysone (20E), essential for molting and reproduction. The synthesis of these ecdysteroids is negatively regulated by MIH, thereby controlling the temporal dynamics of molting cycles [38, 39]. The mandibular organs produce MF, a sesquiterpenoid hormone structurally and functionally analogous to insect juvenile hormones, which regulates multiple physiological processes, including molting, metamorphosis, and ovarian development. The production of MF is under the inhibitory control of MOIH secreted by the XO–SG complex [40, 41]. By adapting to external cues, the endocrine system ensures that reproductive activities occur under favorable conditions, optimizing energy allocation between growth, reproduction, and survival.

### 3.1. Hormonal Regulatory Networks of Ovarian Development in Crustaceans: Functional Models and Potential Applications in Aquaculture

#### 3.1.1. Hormonal Regulation

**Figure 2:**
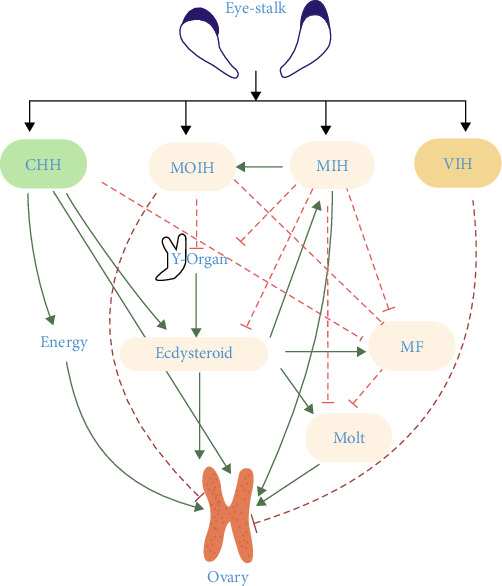
Hormonal regulation of crustacean ovarian development. CHH, crustacean hyperglycemic hormone; MIH, molt inhibiting hormone; MOIH, mandibular organ-inhibiting hormone; VIH, vitellogenesis-inhibiting hormone. Green arrows indicate positive influence, red lines indicate negative influence.

The hormonal regulation of ovarian development in crustaceans represents a complex, multilayered regulatory network that integrates neuroendocrine and endocrine signaling pathways to precisely coordinate vitellogenesis, oocyte maturation, and reproductive development (Figure 2) [42]. This network is governed by an array of hormones synthesized in specialized organs, each executing distinct yet interconnected functions to ensure reproductive success [36]. Here, we systematically analyzed these regulatory hormones across diverse crustacean taxa (Table 1).

The CHH is a multifaceted neuropeptide instrumental in crustaceans. Although its primary function is the regulation of energy metabolism, evidence shows that it is also involved in other important physiological processes, particularly molting and vitellogenesis [44]. Webster [51] reported the presence of CHH receptors in several tissues, including the oocyte membranes of the shore crab (*Carcinus maenas*) and edible crab (*Cancer pagurus*), suggesting that different CHH isoforms may exert specific functions in these sites. In *H. americanus*, CHH isoforms produced in the X-organ are involved in initiating vitellogenesis, with CHH-B specifically responsible for promoting oocyte maturation before spawning [52]. Another study related to the *H. americanus* demonstrated that alternative splicing of the CHH gene produces isoforms that are expressed in the ovary and hepatopancreas, where they regulate vitellogenesis during reproduction [53]. During ovarian maturation in *S. paramamosain*, CHH expression levels significantly increased from stages I to IV, coinciding with active vitellogenesis and oocyte maturation, and declined at stage V once vitellogenesis is complete [54]. These studies indicate that CHH and its isoforms are not only limited to energy metabolism but are also strongly associated with vitellogenesis in crustaceans.

The VIH, also referred to as the gonad-inhibiting hormone (GIH), is synthesized and secreted by neuroendocrine cells of the XO–SG complex located in the eyestalk of many crustacean species [55]. Apart from the XO-SG, this peptide has also been identified in the brain and ventral nerve cord [56]. VIH regulates reproduction in crustaceans by preventing vitellogenesis, either through the suppression of vitellogenesis in the ovary and hepatopancreas or inhibiting vitellogenin (Vg) uptake by oocytes [57]. This inhibition occurs through multiple mechanisms, including the repression of *Vg* expression via the GC/cGMP-MAPK pathway in the hepatopancreas and ovary [58], as well as the activation of the crab adipokinetic hormone/corazonin-related peptide pathway [56]. Functional studies have shown that knocking down VIH results in the upregulation of a suite of reproduction-associated genes, such as phospholipase C and A, COX, estrogen sulfotransferase, prostaglandin E synthase, and short neuropeptide F (sNPF), NPF-1, and NPF-2, all of which contribute to ovarian maturation [56, 59]. In addition, CHH and VIH operate as metabolic counterbalances: CHH mobilizes energy to meet reproductive demands, whereas VIH regulates the initiation of vitellogenesis. This antagonistic relationship manifests temporally through the Vg-phase-specific decline in VIH, which releases ovarian suppression, while CHH concurrently enhances energy bioavailability [55, 60]. The coordinated downregulation of VIH, coupled with CHH-mediated energy mobilization, demonstrates an endocrine-mediated resource allocation trade-off wherein these hormones synchronize reproductive timing and energy allocation.

MIH, primarily recognized for its role in molting, also functions as a regulator of reproduction in crustaceans [58]. In decapod crustaceans, MIH is recognized to have dual functions, inhibiting ecdysteroid secretion from the Y-organs to regulate molting while also stimulating vitellogenesis in the hepatopancreas [61–63]. This was revealed by identifying that MIH-specific membrane binding sites are not only in the Y-organs but also in the hepatopancreas. Notably, the number of these binding sites in the hepatopancreas increases nearly twofold by ovarian stage Ⅲ compared with the earlier stages (I and II) [46]. MIH exerts its effect on the maturing ovary by binding to receptors in the hepatopancreas, which activates the AC/cAMP/PKA signaling pathway and subsequently promotes *Vg* expression [63]. In addition, MIH may also influence the reproductive cycle of crustaceans by suppressing the release of MF from the mandibular organ [64]. Evidence for this was provided by the observed upregulation of juvenile hormone esterase-like carboxylesterase (JHE-like CXE) and farnesoic acid O-methyltransferase (FAMeT) in the ovary following eyestalk ablation. JHE-like CXE functions as a major enzyme for MF degradation, maintaining appropriate MF levels and preventing the harmful effects of excess MF on oocyte development, whereas FAMeT is involved in MF biosynthesis. These results suggest that the loss of MIH caused by eyestalk ablation enhances MF production through increased activity of FAMeT [65]. In summary, MIH performs dual functions that regulate both molting and reproduction in crustaceans. By stimulating Vg production to support oocyte development and modulating MF levels to safeguard ovarian physiology, MIH emerges as an essential determinant of reproductive success.

Although ecdysteroids are traditionally regarded as molting hormones, few studies have shown that they regulate vitellogenesis and drive ovarian maturation in decapod crustaceans [49, 66]. Subramoniam reviewed the functions of ecdysteroids in crustaceans, proposing that after being synthesized in the Y-organs, these hormones accumulate in developing ovaries, possibly through an interaction with Vg. The stored ecdysteroids are believed to act as a reserve for the embryo during external brooding. Thus, ecdysteroids are regarded as key regulators not only of molting but also of reproduction, paralleling their roles in certain insect species [10, 42]. Girish et al. [67] demonstrated that MF acts synergistically with ecdysteroids through the retinoid X receptor (RXR) and the ecdysone receptor (EcR), both of which are central to the induction of vitellogenesis. A recent study reported that in the black tiger shrimp *P. monodon*, EcR also functions as a regulatory factor by suppressing the activity of GIH, a neuropeptide that inhibits vitellogenesis, thereby supporting ovarian maturation [68]. Further systematic investigation into the influence of ecdysteroids on reproduction would provide valuable insights beyond what has been achieved to date.

MF is regarded as the crustacean counterpart of insect juvenile hormone due to their close structural similarity, differing only by the presence of an epoxide moiety in juvenile hormone that regulates metamorphosis. Recent studies emphasize the importance of MF in crustacean reproduction [42]. Experimental administration of MF produced nearly a threefold increase in the ovarian index compared with controls. MF, a sesquiterpene secreted by the mandibular organ [69], is synthesized through the conversion of farnesoic acid into MF by FAMeT in the presence of S-adenosylmethionine (SAM), followed by modification through a cytochrome P450 monooxygenase [70]. After secretion, MF circulates through the hemolymph to target tissues, binding to the RXR in the hepatopancreas and ovary and stimulating vitellogenesis [57]. Deposition of Vg in the ovary enhances ovarian development, marked by increased oocyte diameter, ovarian index, and elevated ovarian protein and lipid content.

#### 3.1.2. Crustacean Ovarian Cyclicity is Governed by an Integrated Neuroendocrine System

The intricate interplay between ecdysteroids and MF, mediated through the neuroendocrine regulation of CHH, MIH, and VIH, underscores the sophisticated hormonal orchestration governing crustacean ovarian cyclicity [38, 39]. In summary, this multifaceted neuroendocrine system coordinates three critical reproductive parameters: (1) metabolic resource allocation through lipid mobilization and hemolymph glucose regulation [60], (2) environmentally-synchronized timing of vitellogenesis progression [71], and (3) phase-specific modulation of oocyte maturation events. Crucially, this system maintains reproductive-quiescent states during unfavorable conditions through VIH-mediated suppression of vitellogenesis while permitting rapid endocrine activation when environmental parameters reach species-specific thresholds [8]. Such dynamic regulation exemplifies the evolutionary sophistication of crustacean reproductive strategies, ensuring precise temporal coordination between energy-intensive vitellogenesis processes, molting cycles, and optimal spawn conditions. The discovery of membrane-bound ecdysteroid receptors mediating nongenomic signaling pathways in ovarian tissue suggests previously unrecognized regulatory mechanisms for rapid oocyte maturation control, challenging existing paradigms of arthropod reproductive endocrinology.

#### 3.1.3. Application on Aquaculture

Building on the established synergistic interactions among CHH, MIH, and MF in regulating ovarian maturation, targeted hormonal supplementation presents a promising strategy for enhancing reproductive efficiency in cultured crustaceans. Empirical evidence from in vivo studies demonstrates that exogenous administration of CHH isoform concentrations induces dose-dependent vitellogenesis in hepatopancreas, while coadministration with MF amplifies oocyte maturation rates [72]. However, species-specific response thresholds necessitate rigorous dose–response characterization, particularly regarding VIH antagonism pathways that could negate provitellogenic effects at supra-physiological concentrations. The implementation of such endocrine modulation strategies could revolutionize crustacean aquaculture by decoupling reproduction from seasonal constraints; however, ecological risk assessments remain imperative for open-system applications. This approach not only addresses global demands for sustainable seafood production but also provides a manipulable model for probing conserved mechanisms of arthropod reproductive physiology.

## 4. Nutritional Regulation of Crustacean Ovarian Development: From Dietary Input to Metabolic Command

Key nutrients and other dietary factors in crustaceans are crucial in successful reproduction and gamete biology. Therefore, providing an optimal broodstock diet is essential to ensure proper gonadal development (Table 2) [85, 86]. As in other aquatic animals, reproduction in crustaceans is highly energy-demanding, with lipids serving as a primary energy source [20]. At certain stages, lipids may account for up to 40% of the total ovarian weight. While proteins and carbohydrates also contribute, lipids are particularly crucial for gonadal maturation and determining egg and larval quality and quantity [87]. The fatty acid composition of crustacean gonads and eggs in wild specimens provides valuable insight into their lipid and fatty acid requirements in aquaculture. In wild-sourced broodstock, elevated levels of unsaturated fatty acids such as arachidonic (20:4n-6), eicosapentaenoic (20:5n-3), and docosahexaenoic (22:6n-3) acids have been observed during ovarian maturation and spawning [88]. Crustaceans have a limited capacity to synthesize key specific nutrients such as proteins, lipids, carbohydrates, and carotenoids, making dietary intake essential. Beyond their dietary supply, however, these nutrients undergo complex regulation within the hepatopancreas and ovary, where they are mobilized, transformed, and integrated into reproductive processes. Consequently, the dual role of nutrients, as both dietary inputs and internal regulators, has become a central focus of research on crustacean reproduction, and will be examined in the following sections as the dietary and regulatory role of nutrients in crustacean ovarian development.

### 4.1. Protein

Proteins form the bulk of crustacean biomass, accounting for 65%–75% of dry weight, and serve as the foundational nutrient for reproductive physiology. Their dietary contribution is essential because they provide the amino acid substrates required for vitellogenesis, oocyte maturation, and embryonic development [86].

#### 4.1.1. Dietary Protein Role

**Figure 3:**
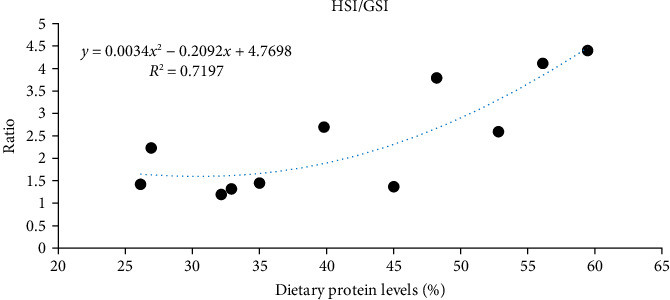
HSI/GSI ratio under different dietary protein levels. HSI/GSI ratio was upregulated as the dietary protein levels increased, which indicates that increased dietary protein levels lead to the accumulation of nutrients in the hepatopancreas. The data reference: [75, 95].

High dietary protein intake demonstrates to improve reproductive performance by advancing oocyte development across various crustacean species [89]. Diets with protein levels ranging from 35.7% to 41.12% promote advanced ovarian stages with a higher frequency of mature oocytes, facilitating yolk protein accumulation and oviposition of red claw crayfish (*Cherax quadricarinatus*) [90]. Similarly, in *L. vannamei*, dietary protein requirements vary from 20% to 50% depending on the physiological stage, with a 52.81% protein diet yielding the highest frequency of mature oocytes and the most favorable ovarian development [91, 92]. On the other hand, arginine, an indispensable amino acid for ovary development, shows a dose-dependent effect: quadratic regression analysis in *E. sinensis* estimated the optimal requirement for adult females at 2.60%, where arginine supplementation decreased VIH content, upregulated *Vg* and *Vg* receptor (*VgR*) expression through the cGMP/cAMP pathway to promote Vg deposition [75]. Mechanistically, arginine supplementation decreased hemolymph VIH concentration, which reduced cGMP and cAMP levels in the hepatopancreas. This downregulation suppressed downstream inhibitory regulators such as PKG and p38-MAPK, while simultaneously activating PKA, a positive regulator of Vg synthesis [93]. Moreover, arginine and other amino acids are known to regulate vitellogenesis via the mTOR signaling pathway, in which Akt acts as a key upstream regulator linking nutrient sensing to yolk protein synthesis [94]. However, Qi et al. [75] reported supraoptimal arginine ( > 2.60%) intake overstimulates the same signaling pathway, elevating VIH secretion and thereby suppressing gonadal growth, which results in a reduced GSI, whereas optimal levels enhance vitellogenesis and ovarian maturation. This connection between dietary protein and amino acid availability underscores the necessity of providing sufficient and optimal protein in broodstock diets to optimize reproductive outcomes. Species-specific thresholds emerge as: (1) dietary 52.81% protein promoted the synthesis of exogenous Vg in female *L. vannamei* [95]; (2) a dietary crude protein content of 32% significantly increased egg area (3.90 mm^2^), volume (39.3 mm^3^), weight (5.44 μg), and diameter (2.27 mm) of redclaw crayfish *C. quadricarinatus* [96]. The protein-reproduction nexus in crustaceans operates through a hepatopancreas-ovary signaling loop, where dietary inputs are transduced into endocrine commands. Precision nutrition strategies that phase protein levels (32%–53%) with vitellogenesis stages, while maintaining specific EAA ratios, could enhance aquaculture yields (Figure 3).

#### 4.1.2. Regulatory Role

Proteins regulate ovarian maturation in crustaceans primarily through their transformation into Vg, a large glycolipophosphoprotein that functions as both a nutrient reserve and a regulatory protein. The hepatopancreas is central to this process, serving not only as a digestive organ but also as the major site of Vg protein synthesis. Once synthesized, Vg is secreted into the hemolymph and transported to the ovary, where it accumulates in the developing oocyte [97]. This regulatory flow intensifies during ovarian maturation; in *E. sinensis*, Vg transfer from the hepatopancreas to the ovary increases significantly between stages II and IV, ensuring sufficient yolk protein accumulation for oocyte growth [98]. The ovary complements this regulation through the expression of *VgR* proteins, which mediate the uptake of Vg. In *L. vannamei*, *VgR* expression peaks during vitellogenesis and declines sharply during embryogenesis [99]. Functional knockdown studies further demonstrate this dependence: suppression of *VgR* expression halts ovarian development at stage II, confirming that Vg-VgR signaling is indispensable for reproduction [100]. Importantly, Vg is not only a storage protein but also a regulatory protein with multifunctional domains. Its N-terminal β-domain facilitates lipid transport into oocytes, while its C-terminal von Willebrand factor domain mediates cortical granule docking during fertilization [101]. Taken together, these findings establish that proteins, through vitellogenesis, transport, and receptor-mediated uptake, function as key molecular signals integrating nutritional status with the endocrine and developmental pathways of crustacean reproduction.

### 4.2. Amino Acid

Crustaceans cannot synthesize essential amino acids (EAAs), so their reproductive success relies on diets with balanced proteins or amino acid supplements [86]. EAAs are critical for Vg and yolk protein synthesis, supporting vitellogenesis, oocyte maturation, and embryonic development. They also act as signaling molecules regulating lipid and energy metabolism within the hepatopancreas–ovary axis [102]. Understanding broodstock amino acid requirements is, therefore, key to promoting ovarian development, fecundity, and larval quality, while supporting sustainable aquaculture practices.

#### 4.2.1. Dietary Role

Dietary arginine at concentrations of 2.52%–3.96% significantly upregulated *Vg* expression in *E. sinensis*, confirming its essential role as an amino acid in vitellogenesis [75]. In *L. vannamei*, dietary arginine supplementation improved ovarian growth and development after a 12-week rearing cycle. At supplementation levels of 4.08% and 4.53%, a greater proportion of females reached ovarian stages III–V, and the GSI was significantly higher than in the control group. This demonstrates a dose-dependent regulatory effect of arginine on ovarian development [74]. Additionally, study in *P. clarkii* demonstrated that an appropriate dietary threonine amount of 16.44 g/kg was found to promote ovarian development [77]. Taurine is often considered an EAA, with many beneficial effects on crustaceans, including growth performance, neuromodulation, gut microbiota, and reproduction. In *P. vannamei*, taurine supplementation 0.6%–1.0% upregulated the *Vg* expression and improved ovarian maturation. It is therefore suggested that an appropriate level of amino acid supplementation is crucial for optimal physiological and reproductive functions of crustaceans.

#### 4.2.2. Regulatory Role

Amino acids have emerged as key functional nutrients regulating ovarian development in crustaceans, acting not only as building blocks for proteins but also as modulators of vitellogenesis, hormone signaling, energy allocation, and oocyte maturation. Currently, limited studies are available on the mechanisms by which amino acids regulate ovarian maturation in crustaceans. In shrimp, type-II VIH (VIH-2) stimulates cGMP production in the hepatopancreas, activating PKG and p38-MAPK to regulate *Vg* expression [93], whereas arginine counteracts this inhibition by reducing VIH release, downregulating the cGMP–MAPK cascade, and activating the cAMP–PKA pathway, thereby promoting Vg synthesis and uptake into oocytes [75]. Arginine also interacts with the mTOR signaling pathway, a key nutrient-sensing regulator of metabolism; increasing dietary arginine has been linked to reduced hepatopancreatic protein content and downregulation of mTOR-related genes [74]. Beyond arginine, threonine demonstrated to enhance vitellogenesis in crayfish by decreasing *MIH* levels and stimulating *Vg* expression via the cAMP–PKA and mTOR pathways. At the same time, taurine supplementation in *P. vannamei* upregulated *IGF-2* expression, which was associated with increased Vg transcription in the hepatopancreas [76]. Further studies are needed to fully elucidate the role of amino acids in regulating ovarian development.

### 4.3. Lipids

Lipids, after protein, are considered the next most important macronutrient in crab nutrition. They include fatty acids and their derivatives, such as triacylglycerols and phospholipids, as well as nonfatty acid lipids like cholesterol and fat-soluble vitamins. Since fatty acids and lipid-based compounds provide nearly twice the energy per unit mass compared to proteins or carbohydrates, they serve as a major energy source in the diets of crustaceans [86].

#### 4.3.1. Dietary Role

Dietary lipids serve not only as a major energy source but also as essential nutrients, performing key roles in crustacean metabolism and contributing to reproductive success by influencing physiological processes such as growth, molting, osmotic balance, and overall health status [103]. Lipids and different classes of fatty acids provide energy to broodstock during ovarian development. *E. sinensis* fed 1% dietary cholesterol showed higher expression of *Vg* and *VgR* gene in hepatopancreas and ovary [104]. Also, a 1.5% cholesterol-fed diet elevated GSI, hepatopancreatic index (HSI), and Vg concentration in the ovary by more nutrient accumulation. When *E. sinensis* was fed a diet with 0.27% cholesterol, the nutrient content in the ovary and hepatopancreas increased significantly, especially cholesterol and lipid, thereby promoting lipid accumulation in the organism [104]. In *L. vannamei*, dietary phospholipid combined with krill oil as a phospholipid source promoted ovarian development [105]. In *S. paramamosain*, moderate dietary arachidonic acid (ARA; n-6 LC-PUFA, 0.46%) promoted the growth and ovary development. Furthermore, Tantikitti et al. [106] reported higher ovarian development in *S. paramamosain* under a combination of astaxanthin (carotenoid) and docosahexaenoic (fatty acid) acid 100 mg/kg and 0.33%, respectively.

#### 4.3.2. Regulatory Role

**Figure 4:**
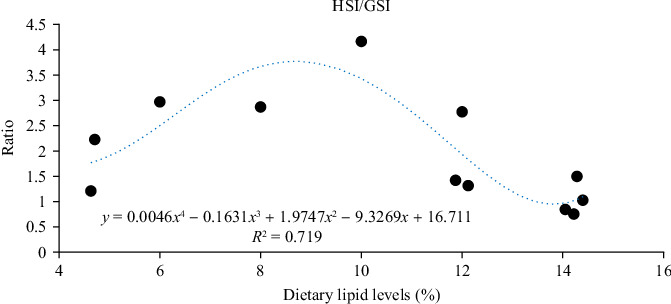
HSI/GSI ratio under different dietary lipid levels. This figure shows a trend of the HSI/GSI ratio first increasing and then decreasing as the dietary lipid levels increase, indicating that lipids exhibit dose-dependent effects on both nutrient accumulation and ovarian development. The data reference: [18, 96, 108–110].

Lipids are fundamental to crustacean reproduction, functioning not only as concentrated sources of metabolic energy but also as structural components of cell membranes, precursors of steroid hormones, and essential drivers of vitellogenesis and oocyte maturation [104]. In *M. rosenbergii* broodstock, dietary lipid levels between 8% and 11.79% significantly enhanced ovarian development, with 10% identified as optimal for maximizing GSI, elevating estrogen and progesterone (PROG) titers, and promoting vitellogenesis; this lipid level also improved antioxidant capacity and upregulated lipid metabolism-related genes such as *FABP* and *DGAT*, thereby sustaining oocyte growth and ovarian integrity [107, 108]. Cholesterol has also been identified as an important dietary component. In *L. vannamei*, a diet containing 1.4% cholesterol enhanced GSI and HSI, upregulated steroidogenic enzymes (17β-HSD and 3β-HSD), and increased circulating estrogen concentration, highlighting its role as a precursor in ovarian steroidogenesis [84]. These findings collectively emphasize that balanced lipid provision is critical for synchronizing hormonal signaling and energy allocation during ovarian maturation. These studies suggest that the proper level of dietary lipids could have a positive effect on regulating ovarian maturation (Figure 4).

In addition to dietary supply, lipid mobilization from storage organs is a defining feature of crustacean reproduction. The hepatopancreas acts as the main lipid reservoir, releasing triglycerides and free fatty acids to the ovary during vitellogenesis. In *S. paramamosain*, triglycerides stored in the hepatopancreas are transported to oocytes to fuel maturation [111]. While in *E. sinensis*, ovarian lipid content rises sharply from stage II to stage IV, dominated by triglycerides and phosphatidylcholine, which together account for over 70% of lipid accumulation before postspawning depletion [13]. A similar redistribution occurs in the giant mud crab (*Scylla serrata*), where hepatopancreatic lipid depletion coincides with ovarian enrichment, reflecting a conserved energy-allocation strategy among crustaceans [88].

Among the lipid classes, LC-PUFAs, such as eicosapentaenoic acid (EPA) and docosahexaenoic acid (DHA), are particularly important for ovarian maturation. These fatty acids are essential for membrane fluidity, vitellogenesis, and embryo development, and their balance in diets directly influences reproductive outcomes [112]. An appropriate n-3/n-6 ratio is especially critical: higher levels of n-3 fatty acids, such as EPA and DHA, have been shown to improve fecundity, egg buoyancy, and larval viability [113, 114]. In *S. paramamosain*, modest n-6 supplementation accelerated vitellogenesis by 4%–16% [115], whereas imbalances or deficiencies delayed ovarian maturation, reduced egg quality, and compromised reproductive success [116]. In *E. sinensis*, DHA supplementation at 0.33% significantly increased GSI, and DHA-enriched eggs showed higher hatching rates and improved larval quality [117]. These findings highlight that LC-PUFA quality, not merely quantity, dictates reproductive success in crustaceans.

### 4.4. Carotenoid

Crustaceans cannot synthesize carotenoids de novo and acquire them directly through their diet [118]. The ovaries are the major part of crustaceans and accumulate a pronounced amount of carotenoids [119], which supplementation can modulate ovary color, immune performance, and antioxidant capacity [120]. Beyond their physiological functions, carotenoids also contribute to flavor quality. They serve as precursors for volatile flavor compounds [121] and, by inhibiting fatty acid oxidation, help maintain both the stability and content of fatty acids [122]. This section synthesizes current knowledge on carotenoid dynamics during ovarian development by focusing on their dietary and regulatory roles.

#### 4.4.1. Dietary Role

Dietary carotenoids (astaxanthin, β-carotene, canthaxanthin, lutein, and zeaxanthin) enhanced the orange–red pigmentation of the ovaries [123]. Appropriate dietary carotenoid concentration in broodstock diets has been demonstrated to improve reproductive performance. Tao et al. [124] reported that in the *M. rosenbergi*, a 0.062% dietary astaxanthin exhibited the highest estradiol level and GSI, and increased Vg levels and ovarian development. In another study, *E. sinensis* were fed with different carotenoids. The results found that canthaxanthin and astaxanthin significantly induced ovarian redness [123]. Moreover, this study also indicated that β-carotene significantly enhanced carotenoid deposition in the ovaries [123]. Beyond coloration and flavor, carotenoids serve as powerful antioxidants for protecting oocytes during maturation by neutralizing oxidative stress [20]. Astaxanthin supplementation also reduced oxidative stress (lower malondialdehyde levels) and concurrently increased ovarian egg diameter and fecundity in narrow-clawed crayfish (*Astacus leptodactylus*) [125]. Besides, very high dietary levels of astaxanthin (0.03%) resulted in its accumulation, together with long-chain PUFAs, in ovarian tissues in white shrimp [126].

#### 4.4.2. Regulatory Role

Carotenoids' content and composition vary significantly throughout crustacean ovarian development [127]. During early maturation, carotenoids accumulate in the hepatopancreas and are transported through the hemolymph to the ovaries during secondary vitellogenesis [128]. The positive effects of astaxanthin are mainly associated with its strong capacity to scavenge oxygen-free radicals and its role in preventing the peroxidation of PUFAs in tissues and diets. In crustaceans, depletion of carotenoids in the hepatopancreas and ovary has been associated with higher superoxide dismutase activity in the hemolymph of captive shrimp compared with wild shrimp [127]. This pattern suggests a limited ability to neutralize oxidative stress during the spawning. Additionally, ovarian development and reproduction are themselves sources of reactive oxygen species, further increasing oxidative pressure [127]. In support of this fact, astaxanthin supplementation at 0.02% exhibited the strongest antioxidant effect, as it significantly reduced malondialdehyde levels in the ovary of narrow-clawed crayfish, *A. leptodactylus*. This reduction was associated with an increase in the number and size of ovarian eggs [125], thereby protecting reproductive tissues and developing eggs from peroxidation damage. Such protection can be attributed to astaxanthin's potent antioxidant capacity to effectively quench excessive singlet oxygen and free radicals generated under various stressors, including ultraviolet radiation, chemical exposure, and physiological stress [129, 130]. Evidence from different crustacean studies suggests that the retinoid derivatives perform a pronounced role in the crustacean developmental process, including larval and ovarian development. Crustaceans contain various retinoids and retinoic acid receptors, and evidence linking these metabolites to enhanced ovarian development in crustaceans highlights their importance in reproductive physiology and successful aquaculture. Since carotenoids are the only source of retinoids in crustaceans, their function as bioactive molecules may have been underestimated [131].

### 4.5. Metabolic Allocation Strategies on Ovarian Development

#### 4.5.1. Hepatopancreatic Energy Allocation and Mobilization Dynamics

Crustacean reproductive success hinges on sophisticated energy partitioning between somatic maintenance and gamete production [132], with the hepatopancreas serving as the central metabolic nexus [133]. During vitellogenesis, the hepatopancreas orchestrates the mobilization of glycogen, lipid, and protein reserves to fuel yolk deposition, a process demanding precise coordination of nutrient flux [134]. Glycogen, primarily stored in the hepatopancreas, ovaries, and muscle tissues, serves as an essential fuel for various metabolic activities, providing the energy needed during reproduction [135]. This polysaccharide reservoir undergoes rapid mobilization for ATP production, which supports the energy-intensive conversion of Vg to yolk proteins. In species like fiddle crab (*Leptuca uruguayensis*), glycogen content in the hepatopancreas decreases significantly during reproductive periods, reflecting its use as a primary energy source for vitellogenesis and the synthesis of yolk proteins and lipids [136]. Biochemical tracking in *L. uruguayensis* reveals cyclical hepatopancreatic glycogen depletion [137], demonstrating evolutionary optimization of glucose metabolism for rapid ATP generation during oocyte maturation. Lipid dynamics exhibit specialized functional compartmentalization: triglycerides provide dense energy reserves while phospholipids serve dual roles in vitellogenesis and membrane biogenesis. The hepatopancreatic lipid-to-glycogen ratio shifts dramatically during reproductive phases, with free fatty acid β-oxidation remarkably increasing during peak vitellogenesis [134]. This metabolic plasticity enables crustaceans to balance the high energy cost of Vg production against maintenance needs [138]. Lipid reserves also play a vital role during reproduction, being crucial for the synthesis of Vg, which is later transformed into lipovitellin, a key yolk protein stored in developing oocytes [133, 139]. This process ensures that sufficient nutrients are available for embryo development after laying eggs, with lipids providing long-term energy storage and proteins acting as building blocks for cell structure and enzyme activity [138].

#### 4.5.2. Reproductive-Phase Energy Flux and Recovery

Energy reserve dynamics in crustaceans exhibit a triphasic pattern throughout the reproductive cycle, characterized by prereproductive buildup, active mobilization during reproduction, and postspawning replenishment. During the prereproductive and reproductive phases, energy reserves in the hepatopancreas are significantly depleted to support the metabolic demands of oogenesis [136]. The blue crab (*Callinectes sapidus*) provides a representative example: lipid droplets, representing a minor component of total lipids in immature ovaries, increase to ~33% during ovarian maturation [140]. During vitellogenesis, hepatopancreatic lipid reserves are mobilized to support ovarian development through lipoprotein-mediated transport [134]. After the reproductive season, energy reserves, particularly glycogen, tend to recover as the metabolic demands of reproduction subside during the postreproductive phase [141]. This cyclical pattern of depletion and recovery highlights the adaptive allocation of resources, allowing crustaceans to balance energy-intensive reproductive processes with the conservation of energy needed for survival during nonreproductive periods.

#### 4.5.3. Metabolic Trade-Offs Between Reproduction and Survival of Crustaceans

The energy dynamic balance in crustacean ovaries involves a complex interplay between storage organs like the hepatopancreas and the metabolic demands of reproductive tissues, alongside the balance between the roles of different energy sources. Lipids and proteins are critical for yolk formation, while glycogen serves as an immediate energy source during oogenesis. Although lipids are considered more energy-dense, glycogen serves as the preferred energy reserve under certain conditions, such as anoxic environments in burrows, due to its rapid mobilization capabilities [85]. However, the energy dichotomy between glycogen (rapid mobilization) and lipids (high-density storage) presents species-specific adaptations: (1) *S. paramamosain* allocates more energy reserves to lipids, capitalizing on their higher caloric yield per unit mass, (2) common shore crab *C. maenas* (L.) maintains higher hepatopancreatic glycogen than lipid stores to survive hypoxic intertidal conditions while reproducing [142]. These delicate balances of energy reserves are essential for the successful reproduction and survival of crustaceans, especially in environments with fluctuating conditions. Furthermore, the synchronization of feeding and reproductive activities ensures that female crustaceans accumulate sufficient energy during the prereproductive period to meet the high energy demands of vitellogenesis and brooding [143, 144].

### 4.6. Nutrients Regulate Ovarian Development by Hormone-Related Pathways

The regulatory interplay between nutritional inputs and endocrine signaling pathways constitutes a fundamental mechanism coordinating crustacean ovarian development, ensuring synchronized vitellogenesis and oocyte maturation. Emerging evidence reveals that specific nutrients exert dual modulation through both metabolic provisioning and endocrine regulation.

#### 4.6.1. Amino Acid-Mediated Endocrine Regulation

Amino acids, particularly L-arginine, serve as central regulators of ovarian development through dual endocrine signaling networks. Dietary arginine demonstrates to enhance vitellogenesis in crustaceans by activating the target of rapamycin (mTOR) signaling pathway, which drives protein synthesis to meet the nutritional and reproductive demands of ovarian maturation [145]. Arginine reduces the inhibitory effects of VIH via the cGMP/PKG and p38-MAPK pathways, further facilitating vitellogenesis, particularly in shrimp and crab [93]. This dual action highlights the capacity of arginine to integrate nutritional and hormonal signals, thereby promoting ovarian maturation.

#### 4.6.2. Lipid-Endocrine Crosstalk

Lipids, especially essential fatty acids, are equally critical for ovarian development by regulating hormones. Cholesterol, a precursor of steroid hormones, plays a pivotal role in integrating nutritional and endocrine pathways during ovarian development. Dietary cholesterol enhanced lipid mobilization and yolk granule deposition in the ovary by increasing the steroid hormone contents of *L. vannamei* [84]. During vitellogenesis, dietary cholesterol enhances the expression of genes involved in steroidogenesis, including low-density lipoprotein receptors (LDLRs) and steroidogenic acute regulatory protein (*STAR*), facilitating hormone synthesis and transport [146]. Cholesterol is metabolized into key hormones, such as PROG and estradiol (E2), which regulate vitellogenesis. In *Portunus trituberculatus*, the upregulation of estrogen-related receptor (ERR) in response to cholesterol supplementation compensates for the absence of classical estrogen receptors, underscoring the central role of cholesterol in ovarian maturation [48, 147]. ARA is an n-6 HUFA with a lower relative proportion in aquatic animals compared with other essential fatty acids [117], necessary for the reproduction of certain aquatic species [148]. A recent study indicated that ARA promotes ovarian development by modulating lipid metabolism and steroid hormone secretion in *E. sinensis* [149].

## 5. Environmental Factors and Their Relationship With Nutrition on Ovarian Development in Crustaceans

### 5.1. The Roles of Environmental Factors in Ovarian Development

**Figure 5:**
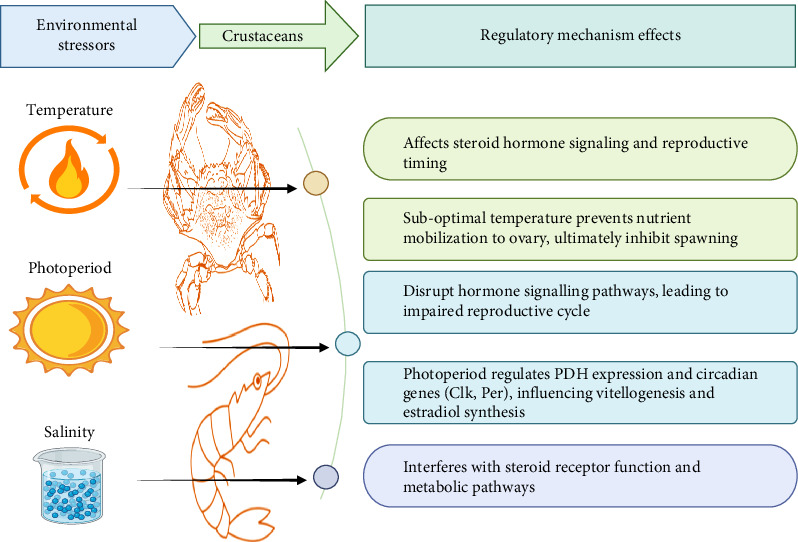
Environmental interactions and regulatory mechanisms in crustaceans. This figure illustrates the dynamic interactions between environmental factors such as light, temperature, and salinity and the regulatory mechanisms that control reproduction and development in crustaceans. It highlights how these environmental factors influence neuroendocrine pathways, affecting hormone secretion and gene expression that govern key processes like vitellogenesis, molting, and ovarian maturation. The integration of these signals ensures proper timing and coordination of reproductive and developmental events in response to external conditions.

Crustacean reproduction is regulated by several hormones, which help assess environmental conditions to determine the optimal time for reproduction [70]. Environmental factors such as salinity, temperature, and photoperiod perform crucial roles in regulating crustacean physiology, including reproduction (Figure 5) [150]. In the following section, knowledge relevant to salinity, temperature, and photoperiod on crustacean reproduction is reviewed.

#### 5.1.1. Salinity

Salinity operates as a vital environmental factor impacting crustacean reproduction, which regulates physiological processes like molting, oogenesis, and embryogenesis [151]. Although comprehensive studies on salinity-mediated ovarian development remain limited, current evidence indicates that species-specific optimal salinity ranges can substantially enhance reproductive performance. For instance, increased salinity positively influences ovarian development and enhances the GSI, which is directly linked to vitellogenesis in *E. sinensis* [152]. This species exhibits reproductive adaptability across a broad salinity spectrum from 8 to 25 ppt, with successful spawning events even under lower salinity conditions [97]. Another study found that reproductive performance in the *E. sinensis* was highest at salinity 18 ppt [153]. In another study, Vazquez et al. [154] reported that shrimps mostly produced eggs and developed their ovaries at all salinities (1, 5, 12, 23, and 34 ppt), but most of the females lost their eggs within 2 days at 5 ppt, whereas successful completion of reproductive events occurred at salinities above 5 ppt. High salinity stimulates broodstock to produce specific substances that induce ovulation [155]. In conclusion, relatively high salinity has a favorable effect on reproduction, but the physiological mechanisms by which salinity promotes mating and ovarian development in crustaceans require further investigation.

#### 5.1.2. Temperature and Photoperiod

Temperature and photoperiod are the key abiotic factors regulating crustacean reproduction, affecting ovarian maturation, spawning, hatching, and larval survival [156]. This is supported by observations in many crustaceans that reproduce seasonally, primarily during long daylengths and warm temperatures in subtropical regions [157]. In some species, the ovary remains in its final stage throughout winter and resumes development once spring begins. This contrasts with crustaceans in equatorial or tropical regions, where spawning occurs more frequently than in subtropical regions due to the extended period of warmer conditions [157]. The effects of temperature and photoperiod may be species-specific but are largely determined by the environmental conditions of their natural habitats. For example, Hidir et al. [158] reported that egg incubation at 26°C is lethal for the crab genus *Scylla* reared in tropical Malaysia, whereas Japanese mud crabs of the same genus can successfully hatch at a much lower temperature of 16.7°C. Additionally, the duration of egg development was considerably shorter when broodstock were reared at higher temperatures. For example, Zeng [159] reported that the berried period of *S. paramamosain* decreased from 29 to 9.5 days. In aquaculture, crustaceans frequently face the problem of egg loss during the berried phase, but elevated temperatures can help mitigate this issue. This was demonstrated by Zeng [159], who showed that hatching and fertilization rates in *S. paramamosain* exceeded 80% under high-temperature conditions. Similarly, Hoang et al. [160] reported that ablated shrimp (*Penaeus merguiensis*) exhibited improved spawning performance at higher temperatures. These studies suggest that raising temperatures to optimal levels promotes reproduction by enhancing ovarian maturation and spawning. This effect may be mediated through elevated estrogen levels, such as estradiol, which stimulate gene expression pathways responsible for ovarian maturation in crustaceans [158]. However, temperatures outside the optimal range, either too low or too high, lead to reduced activity, diminished food intake, and metabolic arrest, thereby preventing nutrient mobilization to the ovaries and ultimately inhibiting spawning in captivity [160].

The photoperiod has been widely investigated for its effects on gonadal development and spawning in crustaceans, as it also influences feeding behavior and activity levels, both of which are closely linked to reproductive performance. For example, extending the photoperiod from 12 to 14 h stimulated gonadal development and increased spawning frequency in the crab *P. pelagicus*. Waddy and Aiken [161] reported that shifting the photoperiod from 8 to 12 h induced spawning in the *H. americanus*, while the same study further demonstrated that prolonged exposure to a 16-h photoperiod promoted synchronized spawning in the same species after 4 months. Similarly, Matsuda et al. [162] observed that spawning occurred in nearly 100% of Japanese spiny lobsters (*Panulirus japonicus*) under long-day photoperiods (14 h), whereas constant light (24 h) completely suppressed spawning. Liu et al. [163] reported similar findings, in which *P. clarkii* requires different light conditions at various ovarian developmental stages, and changes in photoperiod and light intensity may influence its endocrine and circadian clock systems. Interestingly, constant darkness also triggered spawning, but the resulting larvae were of poor quality [164]. In addition, studies on *P. clarkii* [165] and *P. japonicus* [162] reported that maturation and spawning could be induced through the combined influence of temperature and photoperiod. For *P. clarkii*, temperature acted as the primary trigger for the onset of gonadal development, whereas photoperiod regulated the duration of the process [165]. Dubé and Portelance [166] reported that in crayfish (*Orconectes limosus*), the combination of darkness with temperatures between 10–12°C accelerated gonadal maturation, whereas a subsequent temperature increase advanced spawning by up to 3 months compared to natural populations. Although temperature showed the strongest correlation with ovarian development, in natural temperate populations of fiddler crab (*L. uruguayensis*), both photoperiod and temperature positively influenced reproduction [136]. Likewise, López-Uriarte et al. [167] reported that the reproductive cycle of females in natural populations of *Macrobrachium tenellum* was closely correlated with water temperature. Overall, photoperiod has a strong influence on gonadal maturation and spawning in crustaceans, with extended light periods generally promoting reproduction, while constant light or darkness disrupts larval quality. Temperature often acts as the primary trigger, with photoperiod modulating reproductive timing and duration across species.

#### 5.1.3. Implications of Ocean Warming for Reproductive Thermal Thresholds

Warm-water crustaceans generally reach sexual maturity at smaller sizes than their cold-water conspecifics because lower temperatures slow molting frequency and lengthen intermolt periods [168]. These extended intermolt intervals favor energy conservation and allow greater somatic growth between molts, resulting in larger adult body sizes in cold-water populations [169]. A study reported that females from lower sea surface temperature populations matured at larger sizes, reflecting a trade-off between growth and reproduction. In colder environments, maturation is delayed until sufficient energetic reserves have accumulated, ensuring greater reproductive investment at larger relative sizes [170]. Ocean warming also strongly influences egg and embryonic development. For example, in snow crab (*Chionoecetes opilio*) from the eastern Bering Sea, development time from gastrula to hatching decreased by 30% when the temperature increased from −1 to 6°C, with embryos even undergoing a short diapause above 1°C [171]. Laboratory studies on *H. gammarus* showed that a 2°C rise during incubation shortened development time [172]. On the other hand, older populations typically spawn over about 3 months, while warmer populations extend spawning up to 8 months, sometimes with two reproductive periods per year when suitable conditions persist. This extension is made possible by the consistent maintenance of optimal temperatures, enabling continuous reproductive activity [170]. Warmer waters, however, may favor rapid growth and more frequent brood production in smaller females, but at the cost of higher juvenile mortality and reduced size at maturity, as predicted for temperate crab populations [173]. Although this could temporarily increase reproductive output, the combined effects of climate change and additional stressors, such as exploitation, make long-term outcomes difficult to predict [173]. In conclusion, ocean warming extends spawning activity but reduces body size, egg quality, and survival, narrowing the reproductive thermal window and threatening long-term crustacean population stability.

### 5.2. Regulatory Mechanisms of Environmental Factors on Ovarian Development by Governing Hormones

Environmental factors mediate crustacean ovarian development through hierarchical hormonal regulation, wherein photoperiod is the primary modulator of endocrine signaling networks (Figure 5). This environmental-endocrine axis exhibits a dual regulatory capacity of stimulating or suppressing reproductive processes, including vitellogenesis, through conserved molecular pathways, offering critical intervention points for aquaculture optimization [42, 174]. Alterations in photoperiod trigger neuroendocrine secretions that influence the biosynthesis of ovarian function-related hormones, such as VIH, juvenile hormone, and estradiol (E2) [70]. Previous studies indicated that photoperiodic shifts disrupt clock genes and steroid hormone pathways, ultimately reducing reproductive capacity and causing ovarian degradation of *Macrobrachium nipponense* [175]. Environmental cues modulate ovarian maturation through conserved endocrine pathways, including insulin-like signaling, ecdysone, and juvenile hormone pathways, which regulate transcriptional networks governing reproductive plasticity [176]. Crucially, experimental evidence reveals that aberrant light exposure downregulates key enzymes in steroid biosynthesis, such as stearyl-sulfatase and cytochrome P450, which leads to reduced estradiol and vitellogenesis, ultimately causing ovarian degeneration [175]. One mechanism underlying the effect of long photoperiods on ovarian maturation has been identified, which is associated with the expression of pigment-dispersing hormone (PDH) in the eyestalk of *S. paramamosain*. This hormone is secreted by the XO, which subsequently induces the expression of gonadotropic hormones, including follicle-stimulating hormone, that are essential for oocyte maturation. The elevated expression of *PDH* under long photoperiod conditions, maintained at high levels until the end of the vitellogenic phase, provides strong evidence for the role of these peptides in regulating oocyte maturation. Light-sensitive molecular components, including rhodopsin family genes and opsins, demonstrate that photic stimulation regulates neuroendocrine activity through circadian clock modulation, a regulatory mechanism phylogenetically conserved in metazoans. This is comparable to the mammalian system, where light signals received by the retina regulate the suprachiasmatic nucleus, which controls circadian rhythms via hormone signaling [177]. In crustacean ovaries, photoperiodic variations induce upregulation of circadian regulatory genes, including clock (*Clk*) and period (*Per*), highlighting photic regulation of reproductive-circadian coupling. Furthermore, light signals suppress the expression of red pigment-concentrating hormone (*RPCH*) and the estradiol synthesis-related genes within the nervous system through clock gene intermediation [178].

### 5.3. Interplay of Environmental and Nutritional Factors in Ovarian Development

The intricate interplay between environmental parameters and nutritional factors influences ovarian development in crustaceans, with cumulative studies highlighting that these factors jointly shape reproductive success across species [179]. In *S. paramamosain*, low salinity enhances the ovarian accumulation of fatty acids, especially DHA and EPA, though paradoxically accompanied by a reduction in GSI, suggesting a trade-off between environmental adaptation and ovary development [180]. Higher temperatures, as seen in *P. trituberculatus*, accelerate ovarian development, enhancing GSI and nutritional quality of ovarian tissues, particularly crude protein and PUFAs [109]. Elevated temperatures initially inhibit ovarian maturation of *Neocaridina davidi* by inhibiting nutrient transfer to the oocytes. When females are transferred to optimal temperatures, rapid ovarian maturation and spawning occur, demonstrating the impact of thermal conditions on reproductive processes [181]. In *Pontastacus leptodactylus*, total darkness significantly boosts spermatozoa production and GSI, while constant light alters protein and fatty acid levels in the hepatopancreas, further illustrating photoperiodic regulation of reproductive outcomes [182].

## 6. Nutritional Interventions for Ovarian Development in Crustaceans: Efficacy and Economic Feasibility

This section will discuss how specific dietary nutrients, including cholesterol, krill oil, fishmeal, fish oil, and vitamins, contribute to ovarian development and reproductive success in crustaceans. It will also examine the economic feasibility of these nutritional strategies, highlighting both their biological efficacy and their cost-effectiveness within aquaculture systems.

### 6.1. Nutritional Intervention

#### 6.1.1. Cholesterol

In crustacean aquaculture, proper dietary cholesterol supplementation is crucial for optimizing ovary development and reproductive capacity, given the inability of these species to synthesize cholesterol de novo [16]. Empirical studies demonstrate an optimal cholesterol threshold for reproductive enhancement, where 0.4% dietary supplementation significantly enhances Vg content in the ovary, and supraoptimal levels (1.6%) do not yield additional benefits in *E. sinensis* [82]. Besides, long-term dietary administration of 0.27% cholesterol also significantly improved body weight, HSI, and GSI in *E. sinensis*, all of which are key indicators of reproductive performance [104]. *Procambarus clarkia* exhibits maximal growth and ovarian maturation with 10 g/kg cholesterol supplementation [183]. Complementary studies in penaeid shrimp reveal that 1.5% cholesterol inclusion in *L. vannamei* broodstock diets enhanced ovarian development by stimulating nutrient accumulation and steroid hormone synthesis pathways [184]. Based on the above analysis, dietary cholesterol levels between 0.27% and 1.5% consistently support improved ovarian development and reproductive success across various crustacean species.

#### 6.1.2. Krill Oil

Antarctic krill oil demonstrates significant potential for promoting ovarian development in crustaceans with established optimal application dosages and potential regulatory mechanisms in several crustacean species. Enriched with PUFAs, phospholipids, and astaxanthin, krill oil has proven effective in enhancing lipid metabolism and ovarian development in *L. vannamei* (4% krill oil diet) and *C. quadricarinatus* (2% krill oil diet) [105, 110]. Similarly, the 4.5% dietary krill oil supplementation is optimal for stimulating the secretion of reproductive hormones in female *M. rosenbergii* [185], thereby promoting vitellogenesis and ovarian development. Compared to soybean lecithin, 2% dietary krill oil promotes more effective deposition of triacylglycerol and cholesterol in the ovary and hepatopancreas, while maintaining lower levels of low-density lipoprotein cholesterol in the serum. This supplementation further accelerates yolk granule deposition and oocyte maturation more efficiently than soybean lecithin [148]. In *E. sinensis*, krill oil outperforms soybean lecithin and egg yolk lecithin in stimulating vitellogenesis, attributed to its unique phospholipid composition and bioavailability [186]. Therefore, krill oil is an excellent source of phospholipids and can be effectively incorporated into the diets of crustaceans during the prereproductive phase, with the optimal concentration of 2%−4.5%.

#### 6.1.3. Fish Meal and Fish Oil

Nutritional optimization studies in *P. clarkii* demonstrate that modifying the fish meal-to-soybean meal ratio from 1:1 to 1:1.5, combined with 0.06% vitamin C and 3.8% fish oil, enhances ovarian development [187]. This formulation boosts GSI, egg production, and spawning rates, emphasizing the importance of balanced fish meal and fish oil ratios, ensuring adequate vitamin C, and increasing fish oil levels to support ovary maturation and reproductive success. Comparative analyses across decapod species reveal conserved lipid requirements. In *E. sinensis*, brood stock fed a 6% dietary fish oil exhibited higher fecundity than those on diets with lecithin and the control group [188]. The giant tiger prawn *P*. *monodon* fed an 8% fish oil diet produced more eggs and spermatozoa compared to those on a 3% fish oil diet [126].

#### 6.1.4. Vitamin

Vitamin E supplementation demonstrates dose-dependent effects on ovarian development in *M. nipponense*, with optimal doses of 80 mg/kg and 160 mg/kg increasing *Vg* expression, accelerating follicular cell proliferation, and reducing oxidative stress [189]. However, supraoptimal vitamin E supplementation (640 mg/kg) caused structural damage to the ovaries and hepatopancreas, indicating a narrow therapeutic concentration range for vitamin E-mediated ovarian maturation [189]. Similar patterns were observed in other species. In *M. nipponensis*, vitamin E supplementation enhanced ovarian development, while deficiencies in vitamins E, A, and C in *Penaeus japonicus* diets retarded ovarian maturation. Vitamin C supplementation increased GSI, fecundity, and hatching rates of *P. clarkii* brood stock [187]. However, excessive vitamin A (30,000 IU/kg) impaired lipid metabolism, vitellogenesis, and hepatopancreatic antioxidant function, underscoring the importance of careful vitamin management for optimal reproductive health in female crustaceans [190].

In summary, cholesterol supplementation (0.27%–.5%) is highly effective in enhancing ovarian development across various crustacean species, as it directly supports Vg production and reproductive hormone synthesis. Krill oil supplementation (2%–4.5%) further boosts lipid metabolism and ovarian maturation, showing superior efficiency over soybean lecithin. The higher fish oil supplementation (more than 3%) in crustaceans could effectively promote ovarian development. Proper vitamin supplementation can enhance ovarian development and then impact reproductive performance.

### 6.2. Economic Feasibility of Nutritional Diets

Cholesterol is a crucial nutrient for crustaceans because they cannot synthesize it de novo, and a sufficient dietary supply is essential for vitellogenesis and ovarian maturation. However, cholesterol is also one of the most expensive feed additives in aquaculture, and excessive supplementation substantially increases feed costs. To alleviate this constraint, alternative sources, such as phytosterols, have been investigated and shown to partially replace dietary cholesterol while maintaining growth performance in shrimp, offering a cost-effective solution to reduce dependency on purified cholesterol [191, 192]. The inclusion of marine by-products, such as fishmeal, shrimp meal, or squid visceral meal, supports good reproductive performance without the cholesterol supplementation, thereby improving both nutritional and economic efficiency [16].

Krill-derived products such as krill meal and krill oil also offer valuable nutritional attributes, including high-quality protein, phospholipids, omega-3 fatty acids, and astaxanthin, which enhance feed palatability, lipid metabolism, and reproductive output [193]. Nevertheless, the economic feasibility of these ingredients differs markedly. Krill meal generally requires higher inclusion levels to achieve growth benefits, making its use in large-scale aquafeeds cost-prohibitive. In contrast, krill oil is more feasible for targeted supplementation in broodstock diets, where modest inclusion levels are sufficient to produce substantial improvements in vitellogenesis, ovarian development, and hatchery productivity, thereby justifying its higher cost [194]. Fish meal, long regarded as the gold standard protein source in aquafeeds, also poses significant economic challenges. Its price exceeds US$1600 per metric ton, and production requires 4–5 tons of whole fish to yield just 1 ton of meal, resulting in both high costs and environmental unsustainability. Although fish meal improves feed efficiency, nutrient uptake, and growth, global supply is limited (around 4.4 million tonnes in 2016), and reliance on it constrains aquaculture expansion. Consequently, cost-effective alternatives such as soybean meal, poultry by-product meal, algae, and insect-based proteins are increasingly promoted to reduce dependency on capture fisheries while ensuring nutritional balance and maintaining farm profitability [195]. Taken together, the economic feasibility of nutritional supplementation in aquaculture lies in adopting a selective approach: minimizing reliance on costly purified cholesterol by using phytosterols and marine by-products, strategically incorporating krill oil at low levels in broodstock diets while avoiding high-cost krill meal conclusion, and progressively replacing fishmeal with sustainable and affordable protein sources.

## 7. Conclusion

Evidence from diverse crustacean species indicates that the balance between inhibitory and stimulatory hormones strongly influences ovarian development. The VIH hormone consistently emerges as the dominant suppressor of ovarian maturation; reductions in VIH levels, together with stimulatory inputs from MF and ecdysteroids, allow vitellogenesis and oocyte growth to proceed. Nutritional studies demonstrate that lipids have the greatest impact, with cholesterol, LC-PUFAs, EPA, DHA, and ARA directly enhancing vitellogenesis, steroid hormone production, and egg quality. Cholesterol, however, remains a costly feed ingredient, so it is necessary to evaluate phytosterols and marine by-products as feasible alternatives. Among amino acids, arginine shows the most pronounced effect by suppressing VIH secretion and promoting Vg uptake into oocytes, though excessive supplementation can impair reproductive performance. Environmental influences further shape ovarian development, with photoperiod and temperature exerting stronger and more reproducible effects than salinity. Overall, ovarian maturation in crustaceans reflects an interaction among endocrine regulation, nutrient availability, and environmental conditions, each contributing to reproductive efficiency. This highlights the importance of a nutrient–environment synergy model, where balanced dietary inputs (such as cholesterol, LC-PUFAs, and amino acids) are combined with favorable temperature and light conditions to improve ovarian maturation and broodstock performance. Future research should prioritize controlled, multifactorial experiments that directly compare the relative influence of key hormones, nutrients, and environmental variables on ovarian development. Such studies will help define species-specific thresholds and guide the formulation of broodstock diets that are not only biologically effective but also economically viable for large-scale aquaculture.

## Figures and Tables

**Table 1:** Synthesizing, nature, and functionality of crustacean hormones involved in ovarian maturation.

Hormone	Crustacean species	Synthesizing site	Nature	Function	Reference
CHH (crustacean hyperglycemic hormone)	*Homarus americanus* (American lobster)	Eyestalk	Neuropeptide	Carbohydrate metabolism; energy mobilization.	[43]
	*Scylla paramamosain* (mud crab)	Eyestalk	Neuropeptide	Stimulate vitellogenesis and ovary development.	[44]

VIH (vitellogenesis-inhibiting hormone)	*Litopenaeus vannamei* (whiteleg shrimp)	Eyestalk	Neuropeptide	Inhibitory effect in vitellogenesis.	[45]

MIH (molt-inhibiting hormone)	*Callinectes sapidus* (blue crab)	Eyestalk	Neuropeptide	Regulate molting, vitellogenesis.	[46]
	*Metapenaeus ensis* (sand shrimp)	Eyestalk	Neuropeptide	Gonad stimulation.	[47]

Ecdysteroid	*Portunus trituberculatus* (swimming crab)	Y-organ	Steroid	Upsurge of ovarian growth and maturation.	[47]
	*Portunus trituberculatus* (swimming crab)	Ovary and hepatopancreas	Steroid	Increase oocyte size and mature oocyte.	[48]
	*Oziothelphusa senex senex* (freshwater crab)	Ovary and hepatopancreas	Steroid	Escalated ovarian index, ovarian vitellin, and oocyte diameter.	[49]

Methyl farnesoate (MF)	*Oziothelphusa senex senex* (freshwater crab)	Mandibular organ	Sesquiterpenoid	Stimulate ovarian maturation.	[50]

**Table 2:** Species-specific variations in dietary nutritional requirements influencing ovarian development in crustaceans.

Species	Protein/amino acid (%)	Lipid/fatty acid (%)	Reported effects	References
Pacific white shrimp (*Litopenaeus vannamei*)	Arachidonic acid (4.65)	—	Induced early maturation and enhanced reproductive performance.	[73]
Pacific white shrimp (*Litopenaeus vannamei*)	Arginine (2.9, 3.58, 4.08, 4.53, 5.04, 5.55)	—	Dietary arginine supplementation of 4.08%–4.53% enhanced vitellogenesis to promote ovarian development.	[74]
Chinese mitten crab (*Eriocheir sinensis*)	Arginine (1.83, 2.52, 3.26, 3.94, 4.81, 5.52)	—	Dietary arginine at 2.52% promoted ovarian maturation by inhibiting VIH hormone and regulating vitellogenesis via cGMP/cAMP pathway.	[75]
Whiteleg shrimp (*Penaeus vannamei*)	Taurine (0.2, 0.4, 0.6, 0.8, 1.0)	—	Taurine supplementation at 0.4%–0.8% enhanced ovarian development.	[76]
Red swamp crayfish (*Procambarus clarkii*)	Threonine (0.7, 0.9, 1.274, 1.644, 2.083, 2.378)	—	Dietary threonine supplementation around 1.48–1.69 improved ovarian development.	[77]
Red claw crayfish (*Cherax quadricarinatus*)	Protein (32, 26)	Lipid (12, 7)	Higher proportions of postvitellogenic oocytes and enhanced protein deposition in the gonads were recorded at a dietary level of 32% protein and 12% lipid.	[78]
	Crude protein (22, 27, 33, 39, 45)	—	Obtained better gonad development at a dietary concentration of 33% compared to lower or higher ones.	[79]
Giant river prawn (*Macrobrachium rosenbergii*)	—	Lipid (6, 8, 10, 12)	Dietary lipid range of 8%–11% is best for ovarian development.	[80]
Freshwater prawn (*Macrobrachium acanthurus*)	—	Lipid (10, 12.5, 15, 17.5, 20)	Lipid level in the range of 15%–17.5% appears to be optimal for female maturation and egg production.	[81]
	—	Cholesterol (0.4, 1.6)	Inclusion of 0.4% cholesterol in the diet may stimulate vitellogenesis gene and protein expression in the ovary and hepatopancreas, leading to higher nutrient accumulation.	[82]
Swimming crab (*Portunus trituberculatus*)	—	Cholesterol (0.12, 1, 2.5)	Dietary cholesterol at 1% enhanced growth and ovarian development by increasing cholesterol transport and steroid hormone synthesis.	[83]
Pacific white shrimp (*Litopenaeus vannamei*)	—	Cholesterol (0.2, 0.8, 1.4, 2.5, 3.2)	Supplementing the diet with 1.4% cholesterol promotes ovarian development and enhances antioxidant capacity in broodstock.	[84]

## Data Availability

This article is based on data available in published sources, which are cited in the text. No new data were generated or analyzed in this study.

## References

[1] Nanda P. K., Das A. K., Dandapat P. (2021). Nutritional Aspects, Flavour Profile and Health Benefits of Crab Meat Based Novel Food Products and Valorisation of Processing Waste to Wealth: A Review. *Trends in Food Science & Technology*.

[2] Shi X., Lu J., Wu Q. (2019). Comparative Analysis of Growth Performance Between Female and Male Mud Crab *Scylla paramamosain* Crablets: Evidences From a 4-Month Successive Growth Experiment. *Aquaculture*.

[3] Farhadi A., Zhao Q., Tan K. (2025). The Molecular Mechanism of Embryonic Development in Decapod Crustaceans. *Reviews in Aquaculture*.

[4] FAO (2018). *The State of World Fisheries and Aquaculture*.

[5] Farhadi A., Fang S., Zhang Y. (2021). The Significant Sex-Biased Expression Pattern of, *Sp-Wnt4*, Provides Novel Insights Into the Ovarian Development of Mud Crab (*Scylla paramamosain*). *International Journal of Biological Macromolecules*.

[6] Šmuc T., Pucelj M. R., Šinkovec J., Husen B., Thole H., Rižner T. L. (2009). Expression Analysis of the Genes Involved in Estradiol and Progesterone Action in Human Ovarian Endometriosis. *Gynecological Endocrinology*.

[7] Yu Y., Zhang M., Wang D. (2024). Whole Transcriptome RNA Sequencing Provides Novel Insights Into the Molecular Dynamics of Ovarian Development in Mud Crab, *Scylla Paramamosain* After Mating. *Comparative Biochemistry and Physiology Part D: Genomics and Proteomics*.

[8] Chen T., Zhang L. P., Wong N. K., Zhong M., Ren C. H., Hu C. Q. (2014). Pacific White Shrimp (*Litopenaeus vannamei*) Vitellogenesis-Inhibiting Hormone (VIH) Is Predominantly Expressed in the Brain and Negatively Regulates Hepatopancreatic Vitellogenin (VTG) Gene Expression. *Biology of Reproduction*.

[9] Qiao H., Xiong Y., Zhang W. (2015). Characterization, Expression, and Function Analysis of Gonad-Inhibiting Hormone in Oriental River Prawn, *Macrobrachium Nipponense* and Its Induced Expression by Temperature. *Comparative Biochemistry and Physiology Part A: Molecular & Integrative Physiology*.

[10] Subramoniam T. (2000). Crustacean Ecdysteroids in Reproduction and Embryogenesis. *Comparative Biochemistry and Physiology Part C: Pharmacology, Toxicology and Endocrinology*.

[11] Alnawafleh T., Kim B. K., Kang H. E., Yoon T. H., Kim H. W. (2014). Stimulation of Molting and Ovarian Maturation by Methyl Farnesoate in the Pacific White Shrimp *Litopenaeus vannamei* (Boone, 1931). *Fisheries and Aquatic Sciences*.

[12] Li X., Han T., Zheng S., Wu G. (2021). Nutrition and Functions of Amino Acids in Aquatic Crustaceans. *Advances in Experimental Medicine and Biology*.

[13] Wu X., Zhu S., Zhang H. (2020). Fattening Culture Improves the Gonadal Development and Nutritional Quality of Male Chinese Mitten Crab *Eriocheir sinensis*. *Aquaculture*.

[14] Dall W., Smith D. M., Moore L. E. (1995). Carotenoids in the Tiger Prawn *Penaeus esculentus* During Ovarian Maturation. *Marine Biology*.

[15] Feng W., Zhao Z., Wang J., Han T. (2023). Nutrient Composition of Ovary, Hepatopancreas and Muscle Tissues in Relation to Ovarian Development Stage of Female Swimming Crab, *Portunus trituberculatus*. *Animals*.

[16] Kumar V., Sinha A. K., Romano N. (2017). Metabolism and Nutritive Role of Cholesterol in the Growth, Gonadal Development, and Reproduction of Crustaceans. *Reviews in Fisheries Science & Aquaculture*.

[17] Zhang M., Xue Y., Qiu B. (2025). Identification of, *Fruitless1*, Isoforms and Their Molecular Function in Gonadal Development and Courtship in the Mud Crab *Scylla paramamosain*. *Aquaculture*.

[18] Aaqillah-Amr M. A., Hidir A., Noordiyana M. N., Ikhwanuddin M. (2018). Morphological, Biochemical and Histological Analysis of Mud Crab Ovary and Hepatopancreas at Different Stages of Development. *Animal Reproduction Science*.

[19] Lu L., Wang T., Liu A., Ye H. (2025). A Single-Cell Atlas of Crab Ovary Provides New Insights Into Oogenesis in Crustaceans. *Advanced Science*.

[20] Wu Q., Waiho K., Huang Z. (2020). Growth Performance and Biochemical Composition Dynamics of Ovary, Hepatopancreas and Muscle Tissues at Different Ovarian Maturation Stages of Female Mud Crab, *Scylla paramamosain*. *Aquaculture*.

[21] Liu Z., Wu X., Wang W., Yan B., Cheng Y. (2014). Size Distribution and Monthly Variation of Ovarian Development for the Female Blue Swimmer Crab, *Portunus pelagicus* in Beibu Gulf, Off South China. *Scientia Marina*.

[22] Wu X., Liu M., Pan J., Chen H., Zeng C., Cheng Y. (2017). The Ovarian Development Pattern of Pond-Reared Chinese Mitten Crab, *Eriocheir sinensis*, H. Milne-Edwards, 1853. *Crustaceana*.

[23] Sun M., Du X. L., Liu J. Q., Dahms H. U., Wang L. (2018). Histological Analysis of Oogenesis and Ovarian Development of the Freshwater Crab Sinopotamon Henanense. *Tissue and Cell*.

[24] Craveiro C., Soares R., Castro-Neto H. (2023). Ovarian Maturation of *Penaeus subtilis* (Decapoda: Penaeidae): A New Insight to Describe Oocyte Development and Somatic Structures. *Acta Zoologica*.

[25] Sharifian S., Kamrani E., Safaie M., Sharifian S. (2015). Oogenesis and Ovarian Development in the Freshwater Crab *Sodhiana iranica* (Decapoda: Gecarcinucidae) From the South of Iran. *Tissue and Cell*.

[26] Amin-Safwan A., Muhd-Farouk H., Mardhiyyah M. P., Nadirah M., Ikhwanuddin M. (2019). Does Water Salinity Affect the Level of 17β-Estradiol and Ovarian Physiology of Orange Mud Crab, *Scylla olivacea* (Herbst, 1796) in Captivity?. *Journal of King Saud University - Science*.

[27] Saearlee M., Semchuchot W., Vanichviriyakit R., Withyachumnarnkul B., Pongtippatee P. (2024). Oogenesis and Spermatogenesis of the Triploid Black Tiger Shrimp, *Penaeus monodon*. *Trends in Sciences*.

[28] Wang L., Guo Q., Levy T., Chen T., Wu X. (2020). Ovarian Development Pattern and Vitellogenesis of Ridgetail White Prawn, *Exopalaemon carinicauda*. *Cell and Tissue Research*.

[29] Zhong Y., Zhao W., Tang Z. (2021). Comparative Transcriptomic Analysis of the Different Developmental Stages of Ovary in Red Swamp Crayfish *Procambarus clarkii*. *BMC Genomics*.

[30] Tong R., Pan L., Zhang X., Li Y. (2022). Neuroendocrine-Immune Regulation Mechanism in Crustaceans: A Review. *Reviews in Aquaculture*.

[31] Liu J., Zhou T., Wang C., Wang W., Chan S. (2020). Comparative Transcriptomics Reveals Eyestalk Ablation Induced Responses of the Neuroendocrine-Immune System in the Pacific White Shrimp *Litopenaeus vannamei*. *Fish & Shellfish Immunology*.

[32] Huang Z. W., Shi Y. H., Wang A. M., Pan Z., Liu C. S., Gu Z. F. (2019). Effects of Eyestalk Ablation on Growth, Gonadal Development and Body Color of *Cherax quadricarinatus*. *J-Global*.

[33] Knigge T., Leblanc G. A., Ford A. T. (2021). A Crab is Not a Fish: Unique Aspects of the Crustacean Endocrine System and Considerations for Endocrine Toxicology. *Frontiers in Endocrinology*.

[34] Keller R. (1992). Crustacean Neuropeptides: Structures, Functions and Comparative Aspects. *Experientia*.

[35] Chen H. Y., Toullec J. Y., Lee C. Y. (2020). The Crustacean Hyperglycemic Hormone Superfamily: Progress Made in the Past Decade. *Frontiers in Endocrinology*.

[36] Webster S. G., Keller R., Dircksen H. (2012). The CHH-Superfamily of Multifunctional Peptide Hormones Controlling Crustacean Metabolism, Osmoregulation, Moulting, and Reproduction. *General and Comparative Endocrinology*.

[37] Toyota K., Miyakawa H., Hiruta C. (2021). Sex Determination and Differentiation in Decapod and Cladoceran Crustaceans: An Overview of Endocrine Regulation. *Genes*.

[38] Toyota K., Matsushima H., Osanai R., Okutsu T., Yamane F., Ohira T. (2023). Dual Roles of Crustacean Female Sex Hormone During Juvenile Stage in the Kuruma Prawn *Marsupenaeus japonicus*. *General and Comparative Endocrinology*.

[39] Techa S., Chung J. S. (2015). Ecdysteroids Regulate the Levels of Molt-Inhibiting Hormone (MIH) Expression in the Blue Crab, *Callinectes sapidus*. *PLoS One*.

[40] Semchuchot W., Chotwiwatthanakun C., Santimanawong W. (2023). Sesquiterpenoid Pathway in the Mandibular Organ of *Penaeus monodon*: Cloning, Expression, Characterization of PmJHAMT and Its Alteration Response to Eyestalk Ablation. *General and Comparative Endocrinology*.

[41] Wainwright G., Webster S. G., Wilkinson M. C., Chung J. S., Rees H. H. (1996). Structure and Significance of Mandibular Organ-Inhibiting Hormone in the Crab, *Cancer pagurus*: Involvement in Multihormonal Regulation of Growth and Reproduction. *Journal of Biological Chemistry*.

[42] Jayasankar V., Tomy S., Wilder M. N. (2020). Insights on Molecular Mechanisms of Ovarian Development in Decapod Crustacea: Focus on Vitellogenesis-Stimulating Factors and Pathways. *Frontiers in Endocrinology*.

[43] Chang E. S., Prestwich G. D., Bruce M. J. (1990). Amino Acid Sequence of a Peptide With Both Molt-Inhibiting and Hyperglycemic Activities in the Lobster *Homarus americanus*. *Biochemical and Biophysical Research Communications*.

[44] Fu C., Huang X., Gong J., Chen X., Huang H., Ye H. (2016). Crustacean Hyperglycaemic Hormone Gene From the Mud Crab, *Scylla paramamosain*: Cloning, Distribution and Expression Profiles During the Moulting Cycle and Ovarian Development. *Aquaculture Research*.

[45] Kang B. J., Okutsu T., Tsutsui N., Shinji J., Bae S. H., Wilder M. N. (2014). Dynamics of Vitellogenin and Vitellogenesis-Inhibiting Hormone Levels in Adult and Subadult Whiteleg Shrimp, *Litopenaeus vannamei*: Relation to Molting and Eyestalk Ablation. *Biology of Reproduction*.

[46] Zmora N., Trant J., Zohar Y., Chung J. S. (2009). Molt-Inhibiting Hormone Stimulates Vitellogenesis at Advanced Ovarian Developmental Stages in the Female Blue Crab, *Callinectes sapidus*: An Ovarian Stage Dependent Involvement. *Saline Systems*.

[47] Tiu S. H. K., Chan S. M. (2007). The Use of Recombinant Protein and RNA Interference Approaches to Study the Reproductive Functions of a Gonad-Stimulating Hormone From the Shrimp *Metapenaeus ensis*. *FEBS Journal*.

[48] Liu M., Pan J., Liu Z., Cheng Y., Gong J., Wu X. (2018). Effect of Estradiol on Vitellogenesis and Oocyte Development of Female Swimming Crab, *Portunus trituberculatus*. *Aquaculture*.

[49] Swetha C. H., Girish B. P., Reddy P. S. (2016). Elucidation of the Role of Estradiol and Progesterone in Regulating Reproduction in the Edible Crab, Oziothelphusa Senex Senex. *RSC Advances*.

[50] Reddy P. R., Nagaraju G. P. C., Reddy P. S. (2004). Involvement of Methyl Farnesoate in the Regulation of Molting and Reproduction in the Freshwater Crab *Oziotelphusa senex* Senex. *Journal of Crustacean Biology*.

[51] Webster S. G. (1997). High-Affinity Binding of Putative Moult-Inhibiting Hormone (MIH) and Crustacean Hyperglycaemic Hormone (CHH) to Membrane-Bound Receptors on the Y-Organ of the Shore Crab *Carcinus maenas*. *Proceedings of the Royal Society B*.

[52] De Kleijn D. P. V., Janssen K. P. C., Waddy S. L. (1998). Expression of the Crustacean Hyperglycaemic Hormones and the Gonad-Inhibiting Hormone During the Reproductive Cycle of the Female American Lobster *Homarus americanus*. *Journal of Endocrinology*.

[53] Wang C. G., Wang W., Shi L. L., Shen Y. C., Chan S. F. (2022). Alternative Splicing of the Lobster (*Homarus americanus*) Crustacean Hyperglycemic Hormone A and B Genes Produce 2 Protein Variants Involved in Vitellogenin Inhibition. *Frontiers in Marine Science*.

[54] Huang H., Fu C., Chen X., Gong J., Huang X., Ye H. (2015). Molt-Inhibiting Hormone (MIH) Gene From the Green Mud Crab *Scylla paramamosain* and Its Expression During the Molting and Ovarian Cycle. *Aquaculture Research*.

[55] Feijó R. G., Braga A. L., Lanes C. F. C. (2016). Silencing of Gonad-Inhibiting Hormone Transcripts in, *Litopenaeus vannamei*, Females by Use of the RNA Interference Technology. *Marine Biotechnology*.

[56] Saetan J., Duangprom S., Songkoomkrong S. (2023). Potent Ovarian Development as Being Stimulated by Cocktail Hormone in the Female *Scylla olivacea*. *Frontiers in Marine Science*.

[57] Toyota K., Yamamoto T., Mori T. (2023). Eyestalk Transcriptome and Methyl Farnesoate Titers Provide Insight Into the Physiological Changes in the Male Snow Crab, *Chionoecetes opilio*, After Its Terminal Molt. *Scientific Reports*.

[58] Ikhwanuddin M., Lyana N. A., Bakar N. H. A., Jasmani S., Bolong A. M. A. (2012). Stimulation of Ovarian Maturation Using Serotonin (5-Hydroxytryptamine) Hormone on Banana Shrimp, *Fenneropenaeus merguiensis* (De Man, 1888). *World Applied Sciences Journal*.

[59] Duangprom S., Saetan J., Phanaksri T. (2022). Acceleration of Ovarian Maturation in the Female Mud Crab With RNA Interference of the Vitellogenesis-Inhibiting Hormone (VIH). *Frontiers in Marine Science*.

[60] Kang B. J., Bae S. H., Suzuki T., Niitsu S., Wilder M. N. (2019). Transcriptional Silencing of Vitellogenesis-Inhibiting Hormone (VIH) Subtype-I in the Whiteleg Shrimp, *Litopenaeus vannamei*. *Aquaculture*.

[61] Luo X., Chen T., Zhong M. (2015). Differential Regulation of Hepatopancreatic Vitellogenin (VTG) Gene Expression by Two Putative Molt-Inhibiting Hormones (MIH1/2) in Pacific White Shrimp (*Litopenaeus vannamei*). *Peptides*.

[62] Rotllant G., Charmantier-Daures M., De Kleijn D., Charmantier G., Van Herp F. (1995). Ontogeny of Neuroendocrine Centers in the Eyestalk of, *Homarus gammarus*, Embryos: An Anatomical and Hormonal Approach. *Invertebrate Reproduction & Development*.

[63] González M., Betancourt J. L., Rodríguez-Ramos T., Estrada M. P., Carpio Y., Ramos L. (2020). Induction of Spawning in Pacific White Shrimp *Litopenaeus vannamei* (Boone, 1931) by Injection of Its Molt Inhibiting Hormone Isoform II Produced in *E. coli*. *Aquaculture Research*.

[64] Hosamani N., Pamuru R. R., Pamanji S. R. (2016). Natural and Induced (Eyestalk Ablation) Molt Cycle in Freshwater Rice Field Crab Oziothelphusa Senex Senex. *Journal of Aquaculture Research & Development*.

[65] Meng X., Zhang M., Gao B., Lv J., Li J., Liu P. (2020). Integrative Proteomic and MicroRNA Analysis: Insights Into Mechanisms of Eyestalk Ablation-Induced Ovarian Maturation in the Swimming Crab *Portunus trituberculatus*. *Frontiers in Endocrinology*.

[66] Brown M. R., Sieglaff D. H., Rees H. H. (2009). Gonadal Ecdysteroidogenesis in Arthropoda: Occurrence and Regulation. *Annual Review of Entomology*.

[67] Girish B. P., Swetha C. H., Reddy P. S. (2017). Serotonin Induces Ecdysteroidogenesis and Methyl Farnesoate Synthesis in the Mud Crab, *Scylla serrata*. *Biochemical and Biophysical Research Communications*.

[68] Kluebsoongnoen J., Saensuwanna A., Jozghorbani M. (2023). A Possible Role of the Ecdysone Receptor in Modulating Gonad-Inhibiting Hormone Gene Expression in the Black Tiger Prawn, *Penaeus monodon*. *Aquaculture*.

[69] Arath Raghavan S. D., Ayanath A. (2019). Effect of Methyl Farnesoate Administration on Ovarian Growth and Maturation in the Freshwater Crab *Travancoriana schirnerae*. *Egyptian Journal of Aquatic Biology and Fisheries*.

[70] Nagaraju G. P. C. (2011). Reproductive Regulators in Decapod Crustaceans: An Overview. *Journal of Experimental Biology*.

[71] Hussain S., Parmar P., Manohar S., Yadav P. (2017). Effect of Photoperiodism on Ovarian Maturation of Female Freshwater Prawn *Macrobrachium lamarrei* Lamarrei (H. Milne Edwards, 1837). *International Journal of Fisheries and Aquatic Studies*.

[72] Buchi S. R., Vaadala S., Hosamani N., Pamuru R. R., Pamanji S. R. (2016). Regulation of Vitellogenesis by Selected Endocrine Modulators in Crab Oziothelphusa Senex Senex, With Special Reference to Methyl Farnesoate. *Aquaculture Reports*.

[73] Xu H., Zhang Y., Luo K. (2017). Arachidonic Acid in Diets for Early Maturation Stages Enhances the Final Reproductive Performances of Pacific White Shrimp (*Litopenaeus vannamei*). *Aquaculture*.

[74] Zhang X., Yin Y., Fan H., Zhou Q., Jiao L. (1986). Arginine Promoted Ovarian Development in Pacific White Shrimp *Litopenaeus vannamei* Via the NO-SGC-cGMP and TORC1 Signaling Pathways. *Animals: An Open Access Journal From MDPI*.

[75] Qi C., Li J., Huang K. (2024). The Regulatory Effects of Arginine on Ovary Development in the Chinese Mitten Crab *Eriocheir sinensis*. *Aquaculture Reports*.

[76] Mozanzadeh M. T., Bahabadi M. N., Morshedi V. (2024). Effects of Dietary Taurine on Maturation Indices, Antioxidant Capacity, Ovaries Amino and Fatty Acids Profile, and Vitellogenin Gene Transcription Level in, *Penaeus Vannamei*, Female Brooders. *Aquaculture Nutrition*.

[77] Yao H., Cao M., Zhang J. (2024). Dietary Threonine Promoted the Growth and Ovarian Development of the Red Swamp Crayfish (*Procambarus clarkii*). *Aquaculture Nutrition*.

[78] Rodríguez-González H., Hernández-Llamas A., García-Ulloa M., Villarreal H. (2013). Effect of Dietary Protein and Lipid Levels on Gonadal Development of Female Redclaw Crayfish *Cherax quadricarinatus*. *Aquaculture Research*.

[79] Rodríguez-González H., Villarreal H., García-Ulloa M., Hernández-Llamas A. (2009). Evaluation of Practical Diets Containing Different Protein Levels on Gonad Development of Female Redclaw Crayfish *Cherax quadricarinatus*. *Aquaculture Nutrition*.

[80] Song J., Jian Y., Xie Y. (2024). The Dietary Lipid Requirement for Optimal Ovarian Maturation and Overall Health in Female Giant River Prawn, *Macrobrachium rosenbergii*, Broodstock. *Aquaculture Nutrition*.

[81] Hernández-Abad G. Y., Hernández-Hernández L. H., Fernández-Araiza M. A. (2018). Effects of Different Dietary Lipid Concentrations on the Egg Production and Egg Quality Produced by *Macrobrachium acanthurus* Females. *Latin American Journal of Aquatic Research*.

[82] Guo H., Wang M., Wang X. (2022). Effect of Dietary Cholesterol on Ovarian Development of Chinese Mitten Crabs (*Eriocheir sinensis*). *Frontiers in Marine Science*.

[83] Zhu T., Jin M., Xie S. (2022). Transcriptome and Targeted Metabolomics Revealed That Cholesterol Nutrition Promotes Ovarian Development by Regulating Steroid Hormone Metabolism in Swimming Crab. *Aquaculture Reports*.

[84] Liang X., Luo X., Chang T., Han F., Xu C., Li E. (2023). Positive Effects of Optimal Dietary Cholesterol Levels on the Ovary Development and Health of Female Pacific White Shrimp, *Litopenaeus vannamei*, Broodstock. *Aquaculture*.

[85] Alhoshy M., Shehata A. I., Habib Y. J., Abdel-Latif H. M. R., Wang Y., Zhang Z. (2022). Nutrigenomics in Crustaceans: Current Status and Future Prospects. *Fish & Shellfish Immunology*.

[86] Esmaeili N., Ma H., Kadri S., Tocher D. R. (2024). Protein and Lipid Nutrition in Crabs. *Reviews in Aquaculture*.

[87] Wouters R., Lavens P., Nieto J., Sorgeloos P. (2001). Penaeid Shrimp Broodstock Nutrition: An Updated Review on Research and Development. *Aquaculture*.

[88] Alava V. R., Quinitio E. T., De Pedro J. B., Priolo F. M. P., Orozco Z. G. A., Wille M. (2007). Lipids and Fatty Acids in Wild and Pond-Reared Mud Crab *Scylla serrata* (Forsskål) During Ovarian Maturation and Spawning. *Aquaculture Research*.

[89] Dabrowski K., Zhang Y. F., Kwasek K., Hliwa P., Ostaszewska T. (2010). Effects of Protein-, Peptide- and Free Amino Acid-Based Diets in Fish Nutrition. *Aquaculture Research*.

[90] Lu X., Peng D., Chen X. (2020). Effects of Dietary Protein Levels on Growth, Muscle Composition, Digestive Enzymes Activities, Hemolymph Biochemical Indices and Ovary Development of Pre-Adult Red Swamp Crayfish (*Procambarus clarkii*). *Aquaculture Reports*.

[91] Xie S., Wei D., Fang W. (2020). Survival and Protein Synthesis of Post-Larval White Shrimp, *Litopenaeus vannamei*, Were Affected by Dietary Protein Level. *Animal Feed Science and Technology*.

[92] Xu W. J., Pan L. Q. (2014). Evaluation of Dietary Protein Level on Selected Parameters of Immune and Antioxidant Systems, and Growth Performance of Juvenile, *Litopenaeus vannamei*, Reared in Zero-Water Exchange Biofloc-Based Culture Tanks. *Aquaculture*.

[93] Chen T., Ren C., Jiang X. (2018). Mechanisms for Type-II Vitellogenesis-Inhibiting Hormone Suppression of Vitellogenin Transcription in Shrimp Hepatopancreas: Crosstalk of GC/cGMP Pathway With Different MAPK-Dependent Cascades. *PLoS One*.

[94] Liu H., Lin Y., Gu J. (2019). The Increase of Amino Acids Induces the Expression of Vitellogenin After Spinning in the Silkworm *Bombyx mori*. *Journal of Insect Physiology*.

[95] Li M., Zhang X., Jiao L. (2023). Dietary Protein Regulates Ovarian Development Through TOR Pathway Mediated Protein Metabolism in Female *Litopenaeus vannamei*. *Aquaculture Reports*.

[96] Rodríguez-González H., Hernández-Llamas A., Villarreal H., Saucedo P. E., García-Ulloa M., Rodríguez-Jaramillo C. (2006). Gonadal Development and Biochemical Composition of Female Crayfish *Cherax quadricarinatus* (Decapoda: Parastacidae) in Relation to the Gonadosomatic Index at First Maturation. *Aquaculture*.

[97] Li Z., Zhou M., Ruan Y. (2022). Transcriptomic Analysis Reveals Yolk Accumulation Mechanism From the Hepatopancreas to Ovary in the Pacific White Shrimp *Litopenaeus vannamei*. *Frontiers in Marine Science*.

[98] Kiris I. G. A., Eroldoğan O. T., Kır M., Kumlu M. (2004). Influence of Neuropeptide Y (NPY) on Food Intake and Growth of Penaeid Shrimps *Marsupenaeus japonicus* and *Penaeus semisulcatus* (Decapoda: Penaeidae). *Comparative Biochemistry and Physiology Part A: Molecular & Integrative Physiology*.

[99] Tiu S. H. K., Hui J. H. L., He J. G., Tobe S. S., Chan S. M. (2006). Characterization of Vitellogenin in the Shrimp *Metapenaeus ensis*: Expression Studies and Hormonal Regulation of, *MeVg1*, Transcription In Vitro. *Molecular Reproduction and Development*.

[100] Ruan Y., Wong N. K., Zhang X. (2020). Vitellogenin Receptor (VgR) Mediates Oocyte Maturation and Ovarian Development in the Pacific White Shrimp (*Litopenaeus vannamei*). *Frontiers in Physiology*.

[101] Zhang S., Wang S., Li H., Li L. (2011). Vitellogenin, a Multivalent Sensor and an Antimicrobial Effector. *The International Journal of Biochemistry & Cell Biology*.

[102] Zhu Y., Wu J., Leng X. (2020). Metabolomics and Gene Expressions Revealed the Metabolic Changes of Lipid and Amino Acids and the Related Energetic Mechanism in Response to Ovary Development of Chinese Sturgeon (*Acipenser sinensis*). *PLoS One*.

[103] Zhang Y., Yuan Y., Zhang M. (2024). High-Resolution Chromosome-Level Genome of Scylla Paramamosain Provides Molecular Insights Into Adaptive Evolution in Crabs. *BMC Biology*.

[104] Guo H., Hua H., Wang J. (2024). The Role of Cholesterol During the Ovarian Maturation and Lipid Metabolism of Female Chinese Mitten Crab (*Eriocheir sinensis*). *Aquaculture Nutrition*.

[105] Liang X., Xu C., Wang P. (2024). Effect of Dietary Krill Oil Levels on the Regulation of Ovary Development in Pacific White Shrimp (*Litopenaeus vannamei*) Broodstock. *Aquaculture*.

[106] Tantikitti C., Kaonoona R., Pongmaneerat J. (2015). Fatty Acid Profiles and Carotenoids Accumulation in Hepatopancreas and Ovary of Wild Female Mud Crab (*Scylla paramamosain*, Estampador, 1949). *Songklanakarin Journal of Science & Technology*.

[107] Leng X., Zhou H., Tan Q. (2019). Integrated Metabolomic and Transcriptomic Analyses Suggest That High Dietary Lipid Levels Facilitate Ovary Development Through the Enhanced Arachidonic Acid Metabolism, Cholesterol Biosynthesis and Steroid Hormone Synthesis in Chinese Sturgeon (*Acipenser sinensis*). *The British Journal of Nutrition*.

[108] Song J., Jian Y., Xie Y. (2024). The Dietary Lipid Requirement for Ovarian Maturation and Health in Female Giant River Prawn, *Macrobrachium rosenbergii*, Broodstock. *Aquaculture Nutrition*.

[109] Wan L., Wu Y., He J., Zhang D., Shi H., Zhang T. (2024). Effects of Different Water Temperatures on the Ovarian Development and Biochemical Composition of *Portunus trituberculatus* After Mating (Decapoda, Portunidae). *Crustaceana*.

[110] Liang X., Luo X., Lin H. (2022). Effects and Mechanism of Different Phospholipid Diets on Ovary Development in Female Broodstock Pacific White Shrimp, *Litopenaeus vannamei*. *Frontiers in Nutrition*.

[111] Zhu T., He Y., Cao H. (2024). Insights Into Lipid Function for Ovarian Development in the Swimming Crab (*Portunus trituberculatus*): A Comparison of Lipid Mobilization and Deposition in Hepatopancreas and Ovary. *Aquaculture Reports*.

[112] Masoudi Asil S., Abedian Kenari A., Rahimi Miyanji G., Van Der Kraak G. (2017). The Influence of Dietary Arachidonic Acid on Growth, Reproductive Performance, and Fatty Acid Composition of Ovary, Egg and Larvae in an Anabantid Model Fish, Blue Gourami (*Trichopodus trichopterus*; Pallas, 1770). *Aquaculture*.

[113] Ghazali A., Azra M. N., Noordin N. M., Abol-Munafi A. B., Ikhwanuddin M. (2017). Ovarian Morphological Development and Fatty Acids Profile of Mud Crab (*Scylla olivacea*) Fed With Various Diets. *Aquaculture*.

[114] Ying X. P., Yang W. X., Zhang Y. P. (2006). Comparative Studies on Fatty Acid Composition of the Ovaries and Hepatopancreas at Different Physiological Stages of the Chinese Mitten Crab. *Aquaculture*.

[115] Fang J. F., Li Q. (2023). The Effects of Inbreeding on Stress Resistance of the Pacific Oyster *Crassostrea gigas* at Different Temperatures and Salinities. *Marine Biology Research*.

[116] Ravid T., Tietz A., Khayat M., Boehm E., Michelis R., Lubzens E. (1999). Lipid Accumulation in the Ovaries of a Marine Shrimp *Penaeus semisulcatus* (De Haan). *Journal of Experimental Biology*.

[117] Jiang X., Pan K., Yang Y., Shu-Chien A. C., Wu X. (2022). Dietary DHA Oil Supplementation Promotes Ovarian Development and Astaxanthin Deposition During the Ovarian Maturation of Chinese Mitten Crab *Eriocheir sinensis*. *Aquaculture Nutrition*.

[118] Amaya E., Nickell D. (2015). Using Feed to Enhance the Color Quality of Fish and Crustaceans. *Feed and Feeding Practices in Aquaculture*.

[119] Babin A., Moreau J., Moret Y. (2019). Storage of Carotenoids in Crustaceans as an Adaptation to Modulate Immunopathology and Optimize Immunological and Life-History Strategies. *BioEssays*.

[120] Zhang R., Zhang L., Jiang X., Wu X., Wang X. (2024). Effects of Dietary β-Carotene on Color and Flavor Quality of Ovaries in Adult Female Chinese Mitten Crab (*Eriocheir sinensis*). *eFood*.

[121] Yang X., Liu J., Wan P., Guo D., Chen D. W. (2022). Use of Egg Yolk to Imitate Meat Aroma. *Food Chemistry*.

[122] Cvetković D., Marković D. (2011). Beta-Carotene Suppression of Benzophenone-Sensitized Lipid Peroxidation in Hexane Through Additional Chain-Breaking Activities. *Radiation Physics and Chemistry*.

[123] Zhang L., Wu J., Jiang X., Wu X., Wang X. (2025). Different Types of Dietary Carotenoids Improve the Color and Odor Quality of *Eriocheir sinensis*, Ovaries. *Aquaculture and Fisheries*.

[124] Tao M., Wei J., De Cruz C. (2025). Dietary Effects of Astaxanthin on Gonadal Development in Female Broodstock of *Macrobrachium rosenbergii*. *Aquaculture Reports*.

[125] Barim-Oz O., Sahin H. (2017). The Influence of Dietary Antioxidant on Ovarian Eggs and Levels of Vitamin E, C, A, Astaxanthin, β-Carotene and Oxidative Stress in Tissues of Astacus Leptodactylus (Eschscholtz) During Reproduction. *Cellular and Molecular Biology*.

[126] Paibulkichakul C., Piyatiratitivorakul S., Sorgeloos P., Menasveta P. (2008). Improved Maturation of Pond-Reared, Black Tiger Shrimp (*Penaeus monodon*) Using Fish Oil and Astaxanthin Feed Supplements. *Aquaculture*.

[127] Liñán-Cabello M. A., Paniagua-Michel J., Zenteno-Savín T. (2003). Carotenoids and Retinal Levels in Captive and Wild Shrimp, *Litopenaeus vannamei*. *Aquaculture Nutrition*.

[128] Harrison K. E. (1990). The Role of Nutrition in Maturation, Reproduction and Embryonic Development of Decapod Crustaceans: A Review. *Journal of Shellfish Research*.

[129] Britton G. (2008). Functions of Intact Carotenoids. *Carotenoids*.

[130] Elbahnaswy S., Elshopakey G. E. (2024). Recent Progress in Practical Applications of a Potential Carotenoid Astaxanthin in Aquaculture Industry: A Review. *Fish Physiology and Biochemistry*.

[131] Liñán-Cabello M. A., Paniagua-Michel J., Hopkins P. M. (2002). Bioactive Roles of Carotenoids and Retinoids in Crustaceans. *Aquaculture Nutrition*.

[132] Sánchez-Paz A., García-Carreño F., Muhlia-Almazán A., Peregrino-Uriarte A. B., Hernández-López J., Yepiz-Plascencia G. (2006). Usage of Energy Reserves in Crustaceans During Starvation: Status and Future Directions. *Insect Biochemistry and Molecular Biology*.

[133] Girish B. P., Swetha C. H., Reddy P. S. (2014). Hepatopancreas But Not Ovary is the Site of Vitellogenin Synthesis in Female Fresh Water Crab, Oziothelphusa Senex Senex. *Biochemical and Biophysical Research Communications*.

[134] Jimenez A. G., Kinsey S. (2015). Energetics and Metabolic Regulation. *Natural History of Crustacea, Physiology*.

[135] Zhang X., Wang J., Wang C. (2022). The Responses of the Ovary and Eyestalk in *Exopalaemon carinicauda* Under Low Salinity Stress. *Fishes*.

[136] Colpo K. D., López-Greco L. S. (2017). Temperature Influences the Reproduction of Fiddler Crabs at the Southern Edge of Their Distribution. *Invertebrate Biology*.

[137] Marciano A., Colpo K. D., Boy C. C., Greco L. S. L. (2022). Female Energy Dynamics in the Southernmost Fiddler Crab: Mixed Breeding Strategy in *Leptuca uruguayensis*. *Zoology*.

[138] Mourente G., Medina A., González S., Rodríguez A. (1994). Changes in Lipid Class and Fatty Acid Contents in the Ovary and Midgut Gland of the Female Fiddler Crab *Uca tangeri* (Decapoda, Ocypodiidae) During Maturation. *Marine Biology*.

[139] Puengyam P., Tsukimura B., Utarabhand P. (2013). Molecular Characterization of Hepatopancreas Vitellogenin and Its Expression During the Ovarian Development by In Situ Hybridization in the Banana Shrimp *Fenneropenaeus merguiensis*. *Journal of Crustacean Biology*.

[140] Lee R. F., Walker A. (1995). Lipovitellin and Lipid Droplet Accumulation in Oocytes During Ovarian Maturation in the Blue Crab, *Callinectes sapidus*. *Journal of Experimental Zoology*.

[141] Antunes G. F., Do Amaral A. P. N., Ribarcki F. P., Wiilland E. F., Zancan D. M., Vinagre A. S. (2010). Seasonal Variations in the Biochemical Composition and Reproductive Cycle of the Ghost Crab *Ocypode quadrata* (Fabricius, 1787) in Southern Brazil. *Journal of Experimental Zoology Part A: Ecological Genetics and Physiology*.

[142] Heath J. R., Barnes H. (1970). Some Changes in Biochemical Composition With Season and During the Moulting Cycle of the Common Shore Crab, *Carcinus maenas* (L.). *Journal of Experimental Marine Biology and Ecology*.

[143] Zmora N., Trant J., Chan S. M., Chung J. S. (2007). Vitellogenin and Its Messenger RNA During Ovarian Development in the Female Blue Crab, *Callinectes sapidus*: Gene Expression, Synthesis, Transport, and Cleavage. *Biology of Reproduction*.

[144] Brante A., Fernández M., Eckerle L., Mark F., Pörtner H. O., Arntz W. (2003). Reproductive Investment in the Crab Cancer Setosus Along a Latitudinal Cline: Egg Production, Embryo Losses and Embryo Ventilation. *Marine Ecology Progress Series*.

[145] Liang H., Ren M., Habte-Tsion H. M. (2016). Dietary Arginine Affects Growth Performance, Plasma Amino Acid Contents and Gene Expressions of the TOR Signaling Pathway in Juvenile Blunt Snout Bream, *Megalobrama amblycephala*. *Aquaculture*.

[146] Ning Y., Chen S., Li X., Ma Y., Zhao F., Yin L. (2006). Cholesterol, LDL, and 25-Hydroxycholesterol Regulate Expression of the Steroidogenic Acute Regulatory Protein in Microvascular Endothelial Cell Line (BEnd.3). *Biochemical and Biophysical Research Communications*.

[147] Lu Y., Liu M., Gong J., Cheng Y., Wu X. (2018). Effect of Exogenous Estrogen on the Ovarian Development and Gene Expression in the Female Swimming Crab *Portunus trituberculatus* (Miers, 1876) (Decapoda: Brachyura: Portunidae). *Journal of Crustacean Biology*.

[148] Xu C., Yang X., Liang Z. (2023). Evaluation of the Role of Soybean Lecithin, Egg Yolk Lecithin, and Krill Oil in Promoting Ovarian Development in the Female Redclaw Crayfish *Cherax quadricarinatus*. *Aquaculture Nutrition*.

[149] Wang A., Liu X., Xu J. (2025). Arachidonic Acid Promotes Ovarian Development by Modulating Lipid Metabolism and Steroid Hormone Secretion in Chinese Mitten Crab (*Eriocheir sinensis*). *Aquaculture*.

[150] Wang L., Zhu J., Hu M. (2024). Comparative Transcriptome Analysis Reveals Molecular Mechanisms of the Effects of Light Intensity and Photoperiod on Ovarian Development in *Procambarus clarkii* (Girard, 1852). *Comparative Biochemistry and Physiology Part D: Genomics & Proteomics*.

[151] Dildar T., Cui W., Ikhwanuddin M., Ma H. (2025). Aquatic Organisms in Response to Salinity Stress: Ecological Impacts, Adaptive Mechanisms, and Resilience Strategies. *Biology*.

[152] Wang X., Yao Q., Chen D. W. (2022). Effects of Acute Salinity Stress on Osmoregulation, Antioxidant Capacity and Physiological Metabolism of Female Chinese Mitten Crabs (*Eriocheir sinensis*). *Aquaculture*.

[153] Huang X., He L., Tan R. (2022). Effects of Salinity on Reproductive Characteristics and Embryo Quality of *Eriocheir sinensis*. *Aquaculture Research*.

[154] Vázquez M. G., Ituarte R. B., Bas C. C., Spivak E. D. (2013). Effects of Temperature and Salinity on the Ovarian Cycle and the Embryonic Development of the Invasive Shrimp *Palaemon macrodactylus*. *Journal of Crustacean Biology*.

[155] Hou X. L., Mao Q., He L., Gong Y. N., Qu D., Wang Q. (2010). Accessory Sex Gland Proteins Affect Spermatophore Digestion Rate and Spermatozoa Acrosin Activity in *Eriocheir sinensis*. *Journal of Crustacean Biology*.

[156] Habashy M. M., Hassan M. M. S. (2011). Effects of Temperature and Salinity on Growth and Reproduction of the Freshwater Prawn, *Macrobrachium rosenbergii* (Crustacea: Decapoda) in Egypt. *International Journal of Environmental Science & Engineering*.

[157] Lipcius R. N., Herrnkind W. F. (1987). Control and Coordination of Reproduction and Molting in the Spiny Lobster *Panulirus argus*. *Marine Biology*.

[158] Hidir A., Aaqillah-Amr M. A., Mohd-Sabri M. (2022). Effect of Temperature on Sex and Steroid Hormones of Purple Mud Crab, *Scylla tranquebarica* (Fabricius, 1798) During Egg Incubation, Larvae Rearing and Juvenile Production. *Aquaculture Research*.

[159] Zeng C. (2007). Induced Out-of-Season Spawning of the Mud Crab, *Scylla paramamosain* (Estampador) and Effects of Temperature on Embryo Development. *Aquaculture Research*.

[160] Hoang T., Lee S. Y., Keenan C. P., Marsden G. E. (2002). Effect of Temperature on Spawning of Penaeus Merguiensis. *Journal of Thermal Biology*.

[161] Waddy S. L., Aiken D. E. (1992). Seasonal Variation in Spawning by Preovigerous American Lobster (*Homarus americanus*) in Response to Temperature and Photoperiod Manipulation. *Canadian Journal of Fisheries and Aquatic Sciences*.

[162] Matsuda H., Takenouchi T., Yamakawa T. (2002). Effects of Photoperiod and Temperature on Ovarian Development and Spawning of the Japanese Spiny Lobster *Panulirus japonicus*. *Aquaculture*.

[163] Liu S., Gong S., Li J., Huang W. (2013). Effects of Water Temperature, Photoperiod, Eyestalk Ablation, and Non-Hormonal Treatments on Spawning of Ovary-Mature Red Swamp Crayfish. *North American Journal of Aquaculture*.

[164] Bembe S., Liang D., Chung J. S. (2017). Optimal Temperature and Photoperiod for the Spawning of Blue Crab, *Callinectes sapidus*, in Captivity. *Aquaculture Research*.

[165] Daniels W. H., D’Abramo L. R., Graves K. F. (1994). Ovarian Development of Female Red Swamp Crayfish (*Procambarus clarkii*) as Influenced by Temperature and Photoperiod. *Journal of Crustacean Biology*.

[166] Dubé P., Portelance B. (1992). Temperature and Photoperiod Effects on Ovarian Maturation and Egg Laying of the Crayfish, *Orconectes limosus*. *Aquaculture*.

[167] López-Uriarte E., Vega-Villasante F., Wehrtmann I. S. (2020). Reproductive Biology of the Freshwater Shrimp *Macrobrachium tenellum* (Smith, 1871) (Decapoda: Caridea: Palaemonidae) in Mexico. *Journal of Crustacean Biology*.

[168] Johnson D. S., Crowley C., Longmire K., Nelson J., Williams B., Wittyngham S. (2019). The Fiddler Crab, *Minuca pugnax*, Follows Bergmann’s Rule. *Ecology and Evolution*.

[169] Groner M. L., Shields J. D., Landers D. F., Swenarton J., Hoenig J. M. (2018). Rising Temperatures, Molting Phenology, and Epizootic Shell Disease in the American Lobster. *The American Naturalist*.

[170] Monteiro J. N., Bueno-Pardo J., Pinto M., Pardal M. A., Martinho F., Leitão F. (2023). Implications of Warming on the Morphometric and Reproductive Traits of the Green Crab, *Carcinus maenas*. *Fishes*.

[171] Webb J. B., Eckert G. L., Shirley T. C., Tamone S. L. (2007). Changes in Embryonic Development and Hatching in *Chionoecetes opilio* (Snow Crab) With Variation in Incubation Temperature. *The Biological Bulletin*.

[172] Schmalenbach I., Franke H. D. (2010). Potential Impact of Climate Warming on the Recruitment of an Economically and Ecologically Important Species, the European Lobster (*Homarus gammarus*) at Helgoland, North Sea. *Marine Biology*.

[173] Hines A. H., Johnson E. G., Darnell M. Z., Krus G. H., Eckert G. L., Foy R. J. (2009). Predicting Effects of Climate Change on Blue Crabs in Chesapeake Bay. *Biology and Management of Exploited Crab Populations Under Climate Change*.

[174] Hong M., Li N., Li J. (2019). Adenosine Monophosphate-Activated Protein Kinase Signaling Regulates Lipid Metabolism in Response to Salinity Stress in the Red-Eared Slider Turtle *Trachemys scripta* Elegans. *Frontiers in Physiology*.

[175] Fu C., Li F., Wang L., Li T. (2019). Molecular Insights Into Ovary Degeneration Induced by Environmental Factors in Female Oriental River Prawns *Macrobrachium nipponense*. *Environmental Pollution*.

[176] Liu J., Zhou T., Wang C., Chan S., Wang W. (2021). Deciphering the Molecular Regulatory Mechanism Orchestrating Ovary Development of the Pacific Whiteleg Shrimp *Litopenaeus vannamei* Through Integrated Transcriptomic Analysis of Reproduction-Related Organs. *Aquaculture*.

[177] Chen X., Zhang W., Gu Y., Huang S. (2025). Circadian Clocks and Their Role in Kidney and Eye Diseases Across Organ Systems. *Frontiers in Physiology*.

[178] Fu C., Li F., Wang L., Wang A., Yu J., Wang H. (2019). Comparative Transcriptology Reveals Effects of Circadian Rhythm in the Nervous System on Precocious Puberty of the Female Chinese Mitten Crab. *Comparative Biochemistry and Physiology Part D: Genomics & Proteomics*.

[179] Waqas W., Yuan Y., Ali S. (2024). Toxic Effects of Heavy Metals on Crustaceans and Associated Health Risks in Humans: A Review. *Environmental Chemistry Letters*.

[180] Han W., Liu H., Wang Y. (2024). Changes of Nutrient Composition in the Ovaries and Hepatopancreas of Mud Crab, *Scylla paramamosain*, Broodstock and Their Offspring Performance at Different Salinities. *Aquaculture*.

[181] Baliña S., Temperoni B., Greco L. S. L., Tropea C. (2018). Losing Reproduction: Effect of High Temperature on Female Biochemical Composition and Egg Quality in a Freshwater Crustacean With Direct Development, the Red Cherry Shrimp, Neocaridina Davidi (Decapoda, Atyidae). *The Biological Bulletin*.

[182] Farhadi A., Harlıoğlu M. M. (2019). Photoperiod Affects Gamete Production, and Protein and Lipid Metabolism in Male Narrow-Clawed Crayfish Pontastacus Leptodactylus (Eschscholtz, 1823). *Animal Reproduction Science*.

[183] Hou S., Zhu S., Li J., Huang J., Li J., Cheng Y. (2022). Effects of Dietary Phospholipid and Cholesterol Levels on Growth, Molting Performance, and Ovary Development in Female Juvenile Crayfish (*Procambarus clarkii*). *Aquaculture Nutrition*.

[184] Ma Q., Wang Z., Xu H., Wei Y., Liang M. (2024). Effects of Dietary Cholesterol on Ovary Development and Reproductive Capacity in Pacific White Shrimp Broodstock, *Litopenaeus vannamei*. *Aquaculture Reports*.

[185] Zheng X., Yang J., Liu X. (2024). Effects of Different Levels of Antarctic Krill Oil on the Ovarian Development of *Macrobrachium rosenbergii*. *Animals*.

[186] Lin Z., Qi C., Han F. (2020). Selecting Suitable Phospholipid Source for Female *Eriocheir sinensis* in Pre-Reproductive Phase. *Aquaculture*.

[187] Xu J. Y., Wang T. T., Wang Y. F., Peng Y. (2010). Effect of Combined Fish Meal: Soybean Meal Ratio, Vitamin C and Fish Oil Supplementations in Diet on the Growth and Reproduction of Red Swamp Crayfish, *Procambarus clarkii* (Crustacea: Decapoda). *Aquaculture Research*.

[188] Liu L. H., Chen L. Q., Li K., Zhou Y. K., Li E. C. (2007). Effects of Dietary Lipid Sources on Ovary Development and Reproduction Performance of Female *Eriocheir sinensis*. *Journal of Fishery Sciences of China*.

[189] Li Y., Fan B., Huang Y., Wu D., Zhang M., Zhao Y. (2018). Effects of Dietary Vitamin E on Reproductive Performance and Antioxidant Capacity of *Macrobrachium nipponense* Female Shrimp. *Aquaculture Nutrition*.

[190] Huang Q., Wang X., Bu X. (2022). Role of Vitamin A in the Ovary Development for Female *Eriocheir sinensis* in the Gonadal Development Stage. *Aquaculture*.

[191] Guo J., Hussain A. S., Tacon A. G. J. (2020). Cholesterol Requirement and Phytosterols Efficiency in Semi-Purified Diets of Juvenile Pacific White Shrimp *Litopenaeus vannamei*. *Aquaculture Nutrition*.

[192] Chen Y., Mitra A., Rahimnejad S. (2024). Retrospect of Fish Meal Substitution in Pacific White Shrimp (*Litopenaeus vannamei*) Feed: Alternatives, Limitations and Future Prospects. *Reviews in Aquaculture*.

[193] Panteli N., Kousoulaki K., Antonopoulou E. (2025). Which Novel Ingredient Should be Considered the “Holy Grail” for Sustainable Production of Finfish Aquafeeds?. *Reviews in Aquaculture*.

[194] Jiang Z., Xu C., Yang X., Han F., Chi M., Li E. (2025). Analysis of the Optimum Krill Oil Supplementation Strategy for Ovarian High-Quality Development in the Female Redclaw Crayfish *Cherax quadricarinatus*. *Aquaculture Reports*.

[195] Hodar A. R., Vasava R. J., Mahavadiya D. R., Joshi N. H. (2020). Fish Meal and Fish Oil Replacement for Aqua Feed Formulation by Using Alternative Sources: A Review. *Journal of Experimental Zoology India*.

